# All-analog photoelectronic chip for high-speed vision tasks

**DOI:** 10.1038/s41586-023-06558-8

**Published:** 2023-10-25

**Authors:** Yitong Chen, Maimaiti Nazhamaiti, Han Xu, Yao Meng, Tiankuang Zhou, Guangpu Li, Jingtao Fan, Qi Wei, Jiamin Wu, Fei Qiao, Lu Fang, Qionghai Dai

**Affiliations:** 1 Department of Automation, Tsinghua University, Beijing, China; 2 Department of Electronic Engineering, Tsinghua University, Beijing, China; 3 Beijing National Research Center for Information Science and Technology, Tsinghua University, Beijing, China; 4 Shenzhen International Graduate School, Tsinghua University, Shenzhen, China; 5 Department of Precision Instruments, Tsinghua University, Beijing, China; 6 Institute for Brain and Cognitive Sciences, Tsinghua University, Beijing, China

**Keywords:** Optoelectronic devices and components, Computer science, Electrical and electronic engineering

## Abstract

Photonic computing enables faster and more energy-efficient processing of vision data^1–5^. However, experimental superiority of deployable systems remains a challenge because of complicated optical nonlinearities, considerable power consumption of analog-to-digital converters (ADCs) for downstream digital processing and vulnerability to noises and system errors^1,6–8^. Here we propose an all-analog chip combining electronic and light computing (ACCEL). It has a systemic energy efficiency of 74.8 peta-operations per second per watt and a computing speed of 4.6 peta-operations per second (more than 99% implemented by optics), corresponding to more than three and one order of magnitude higher than state-of-the-art computing processors, respectively. After applying diffractive optical computing as an optical encoder for feature extraction, the light-induced photocurrents are directly used for further calculation in an integrated analog computing chip without the requirement of analog-to-digital converters, leading to a low computing latency of 72 ns for each frame. With joint optimizations of optoelectronic computing and adaptive training, ACCEL achieves competitive classification accuracies of 85.5%, 82.0% and 92.6%, respectively, for Fashion-MNIST, 3-class ImageNet classification and time-lapse video recognition task experimentally, while showing superior system robustness in low-light conditions (0.14 fJ μm^−2^ each frame). ACCEL can be used across a broad range of applications such as wearable devices, autonomous driving and industrial inspections.

## Abstract (web summary)

An all-analog chip combining electronic and light computing achieves systemic energy efficiency of more than three orders of magnitude and a computing speed of more than one order of magnitude compared with state-of-the-art computing processors.

## Main

Computer vision has broad applications, including autonomous driving^9,10^, robotics^11^, medical diagnosis^12–14^ and wearable devices^15,16^. Although deep learning has notably improved the performance of vision tasks at the algorithmic level^17,18^, these tasks are fundamentally limited by energy consumption and computing speed of traditional digital computing units. During a typical vision task, a high-resolution image is first captured by the sensor, then digitized by a large number of analog-to-digital converters (ADCs) and processed through a neural network (NN) on a digital processing unit for classification. In this case, high-throughput, high-precision ADCs reduce the imaging frame rate because of limited data bandwidth and lead to considerable energy consumption. Moreover, short exposure time is required to complete vision tasks with ultra-low latency, demanding extremely high computing power and noise robustness.

Recently, photonic computing has emerged as one of the most promising approaches to address these problems^1–5,19^. It uses the features of light to represent information and to compute using propagation and interference^1,2,5,6,20–32^. By implementing deep neural networks (DNNs), optical neural networks (ONNs) have been reported to achieve a computing efficiency of 1.58 tera-operations per second (TOPS) per watt^5–7^, much higher than advanced digital electronic computing platforms such as graphic processing units (GPUs)^33,34^ (about 0.52 TOPS W^−1^). However, existing photonic computing systems still suffer from severe practical limitations, including complicated implementation of optical nonlinearity, considerable power consumption of ADCs and vulnerability to noises and system errors. For example, Mach–Zehnder interferometers are usually constrained by integration scales from achieving high systemic computing speed^7^, whereas diffractive DNNs with abundant nodes are hard to incorporate efficient optical nonlinearity^1,6^. Moreover, previous ONNs may be sensitive to noise at a low signal-to-noise ratio (SNR)^8,28,35^, making them vulnerable to shot-noise fluctuations because of ultra-short exposure time. These issues notably prevent existing photonic computing from demonstrating systemic supremacy over traditional digital computing in practical computer vision tasks.

Here we propose an all-analog chip combining electronics and light, named ACCEL, for energy-efficient and ultra-high-speed vision tasks with competitive task performance and scalability. Instead of turning to digital units to tackle optical computing limitations, ACCEL fuses diffractive optical analog computing (OAC) and electronic analog computing (EAC) with scalability, nonlinearity and flexibility in one chip. In this way, ACCEL achieves an experimental energy efficiency of 74.8 peta-OPS W^−1^ and a computing speed of 4.6 peta-OPS, three and one order of magnitude higher than state-of-the-art computing chips, respectively. To compensate for manufacturing defects and alignment errors, we develop an adaptive training method, leading to experimental test accuracies of 97.1%, 85.5% and 74.6% over the 10-class classification of MNIST (Modified National Institute of Standards and Technology), Fashion-MNIST and Kuzushiji-MNIST (KMNIST), respectively, as well as 82.0% on 3-class ImageNet classification and 92.6% on 5-class traffic video judgement. By conducting noise-robust feature extraction with OAC, ACCEL reduces massive sampling requirements during photoelectric conversion with robustness under ultra-low exposure (about 0.14 fJ μm^−2^ per frame), achieving up to 29.4% increase of accuracy compared with individual optical or electronic NNs. Furthermore, ACCEL can be reconfigured for different tasks by EAC without changing the OAC module. We believe that the marked performance of ACCEL demonstrates a practical solution for next-generation intelligent computing by including the advantages of both photons and electrons in an all-analog way.

## The architecture of ACCEL

**Fig. 1:**
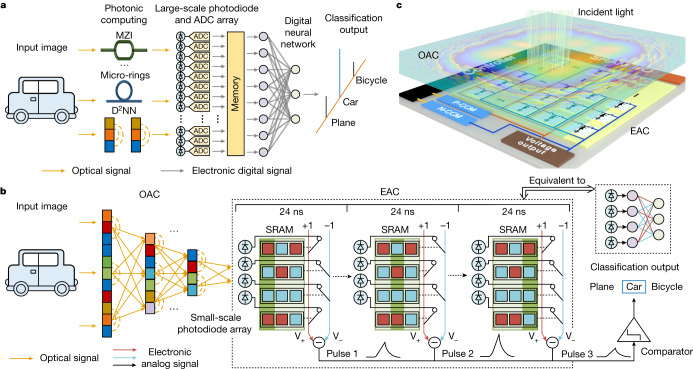
The architecture of ACCEL. **a**, The workflow of traditional optoelectronic computing, including large-scale photodiode and ADC arrays. **b**, The workflow of ACCEL. A diffractive optical computing module processes the input image in the optical domain for feature extraction, and its output light field is used to generate photocurrents by the photodiode array for analog electronic computing directly. EAC outputs sequential pulses corresponding to multiple output nodes of the equivalent network. The binary weights in EAC are reconfigured during each pulse by SRAM, by switching the connection of the photodiodes to either V_+_ or V_−_ lines. The comparator outputs the pulse with the maximum voltage as the predicted result of ACCEL. **c**, Schematic of ACCEL with an OAC integrated directly in front of an EAC circuit for high-speed, low-energy processing of vision tasks. MZI, Mach–Zehnder interferometer; D^2^NN, diffractive deep neural network .

As digital devices remain the mainstream, vision tasks usually require to convert the optical signals even after optical computing into digital signals by large-scale photodiodes and power-hungry ADCs for necessary post-processing (Fig. 1a). Otherwise, complicated implementation of precise optical nonlinearity and memory are required, usually at the cost of latency and power consumption at the system level^36–39^. Here we design an optoelectronic hybrid architecture in an all-analog way to reduce massive ADCs for high-speed and power-efficient vision tasks with competitive task performance. By illuminating targets with either coherent or incoherent light, we encode the information into light fields. With a common imaging system, ACCEL is placed at the image plane for direct image processing such as classifications. The first part of ACCEL interacting with the light field is a multi-layer diffractive optical computing module^1^ to extract features of high-resolution images at light speed, termed as OAC, reducing the requirement of optoelectronic conversion through dimension reduction all-optically (Fig. 1b). Phase masks in OAC are trained to process the data encoded in light fields with operations of dot product and light diffraction, equivalent to linear matrix multiplications of a complex light field. The extracted features encoded in light fields after OAC are connected to EAC with a 32 × 32 photodiode array to convert optical signals into analog electronic signals based on the photoelectric effect, serving as a nonlinear activation. Without the requirement of ADCs, these photodiodes are either connected to the V_+_ positive line or V_−_ negative line determined by the weights stored in static random-access memory (SRAM). The generated photocurrents are first summed up on both lines based on Kirchhoff’s law. Then an analog subtractor calculates the differential voltage of the computing lines V_+_ and V_−_ as an output node. By resetting the computing lines and updating weights with SRAM, ACCEL can output another pulse with different connections of photodiodes. Therefore, EAC is equivalent to a binary-weighted fully connected NN, corresponding to the calculation matrix of 1,024 × *N*_output_ (where *N*_output_ is the number of output pulses). The output can be directly used as predicted labels of classification categories or as inputs of another digital NN. For all-analog computation, we set *N*_output_ as *n* for *n*-class classification without any digital NNs. For ACCEL with a single EAC core, it works sequentially by outputting multiple pulses corresponding to *N*_output_ output nodes of the binary NN in EAC (Fig. 1b). All these functions can be integrated on one chip in an all-analog way for broad applications and are compatible with existing digital NNs for more complicated tasks (Fig. 1c).

**Fig. 2:**
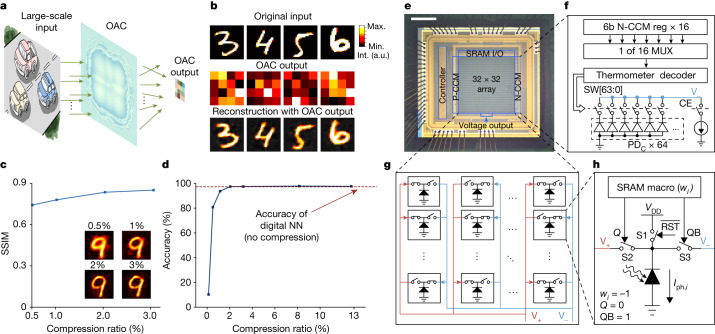
Implementation of ACCEL. **a**, The principle of OAC for feature extraction of large-scale images. **b**, Simulated examples of OAC processing. OAC encodes the 28 × 28 original inputs into 4 × 4 features. A three-layer fully connected digital NN (Supplementary Table 1) reconstructs the image with the OAC output features. **c**, The SSIM (structural similarity index) of reconstruction results with OAC outputs under different compression ratios obtained by numerical simulations on the MNIST dataset. Examples of reconstruction images corresponding to different compression ratios are displayed in the corner. Compression ratio is the ratio of the dimensionality of OAC output to the dimensionality of original images. The example images for the original input are adapted from the MNIST dataset^40^ with permission. **d**, Classification accuracy by using OAC output as the input connected to a three-layer fully connected digital NN (Supplementary Table 1) under different compression ratios of OAC obtained by numerical simulations. The pixel size of the phase mask in OAC is 3 µm and the diffraction distance is 3 mm. The neuron number in OAC is 500 × 500. The red dashed line is the classification accuracy of the digital NN using the original images without OAC as the input. **e**, Photo of the EAC chip. Scale bar, 500 μm. The chip consists of a 32 × 32 photodiode array, two capacitance compensation modules P-CCM and N-CCM, voltage output module and peripheral SRAM I/O and controller. **f**, The structure of the capacitance compensation module. **g**, The structure of the EAC array. **h**, Magnified circuit structure of each pixel. a.u., arbitrary unit; Max., maximum; Min., minimum; Int., intensity; PD, photodiode. Source Data

For OAC, we integrated diffractive optical computing directly in front of EAC with a specific distance to conduct feature extraction as an optical encoder (Fig. 2a). Weights in phase masks can be trained with numerical beam propagations based on Rayleigh–Sommerfeld diffraction theory. A simple three-layer digital NN (Supplementary Table 1) can reconstruct images in the MNIST dataset with only 2% samplings, demonstrating the data compression performance of OAC (Fig. 2b,c). Furthermore, when directly using a digital NN (Supplementary Table 1) for classification with the output of OAC, the same classification accuracy can be achieved with reduced samplings (Fig. 2d). Thus, the number of ADCs can be effectively reduced by 98% without impairment on accuracy. Addressing more complicated tasks or being connected to a less complicated network may reduce the compression rate and require higher dimensionality for the feature space.

**Extended Data Fig. 1:**
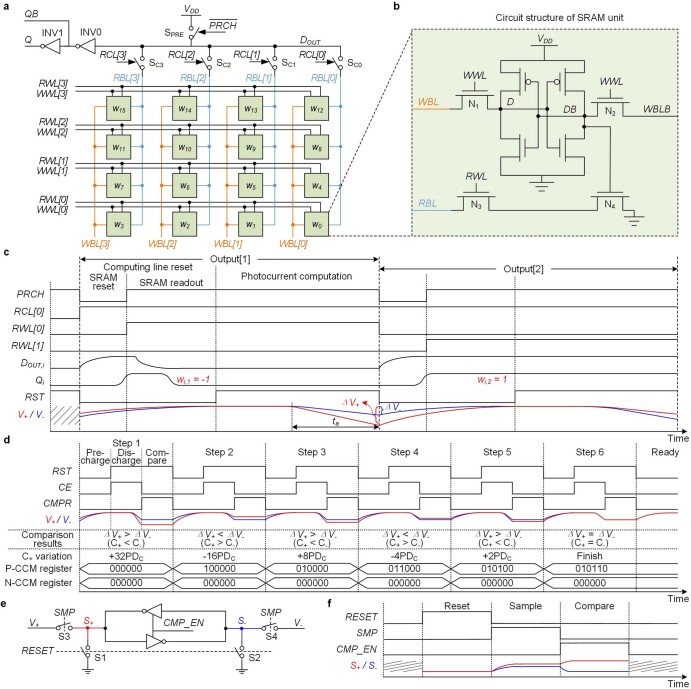
Implementation and timing diagrams of EAC circuit. **a**,**b**, Implementation of the SRAM macro in each pixel. The SRAM unit circuit is implemented with the standard 8-transistor structure. RWL, read word-line; WWL, write word-line; RBL, read bit-line; RCL, read-column; PRCH, pre-charge. **c**, Timing diagram of the signals in the EAC chip during computation, which consists of four operations: SRAM reset, SRAM readout, computing line reset and photocurrent computation. Signal *D*_OUT,__*i*_ is the internal pre-charging node in the SRAM macro in *i*_th_ pixel unit (1 ≤ *i* ≤ 1024). Signal *Q*_*i*_ is the output of SRAM macro in *i*_th_ pixel unit, determined by the stored weight *w*_*ij*_, where 1 ≤ *j* ≤ *N*_output_ labels the *j*_th_ output node. The SRAM operation and reset operation of computing line can be conducted simultaneously. **d**, Timing diagram of the capacitance compensation process. The compensation process is performed by a binary search strategy in multiple steps to tune the load capacitance C_+_/C_−_ of the computing line V_+_/V_−_ to the same value. The presented 6-bit binary codes stored in the P-CCM/N-CCM registers indicate the number of the compensation photodiodes (PD_C_) in the P-CCM/N-CCM connected to the computing line. The variations of C_+_ and P-CCM/N-CCM registers illustrated in this figure correspond to the case where the number of positive/negative weights are 490/534. **e**, The circuit structure of the comparator. The comparator utilizes back-to-back inverters that form a latch for comparing and switches for timing controlling. **f**, Timing diagram of the comparator. The operation of the comparator includes three phases: reset (RESET), sample (SMP) and compare (CMP_EN).

For EAC, we have 32 × 32 pixel circuits here (Fig. 2e–g), corresponding to the calculation matrix of 1,024 × *N*_output_ with the weight *w*_*ij*_, where 1 ≤ *i* ≤ 1,024 labels the *i*th photodiode, and 1 ≤ *j* ≤ *N*_output_ labels the *j*th output node (voltage pulse) with a maximum number *N*_output_ = 16 in our fabricated chip. Each pixel circuit is composed of one photodiode to generate photocurrent *I*_ph*,i*_ used directly for analog computing, three switches and one SRAM macro to store weights *w*_*ij*_ of the binary network (Fig. 2h, Extended Data Fig. 1 and Supplementary Note 1). By turning on either switch S2 or S3 with the SRAM macro, determined by the weight *w*_*ij*_, the cathode of the *i*th photodiode is connected to the positive computing line V_+_ (*w*_*ij*_ = 1) or negative computing line V_−_ (*w*_*ij*_ = −1) for the *j*th output node. The on-chip controller writes trained weights to SRAM macro in each pixel through SRAM input/output (I/O) before inference. The accumulated photocurrents with either positive or negative weights discharge the computing lines. The voltage-drop difference between V_+_ and V_−_ after an accumulating time *t*_a_ is sent out directly as an output pulse (Methods). The computing power consumption of EAC mainly comes from the discharging power of the photocurrent. Meanwhile, all pixels compute simultaneously, thus not only improving computing speed but also reducing readout noises. The voltage-drop difference of the *j*th output node between computing lines V_+_ and V_−_ proportionally correspond to the computation of Δ*V*_*j*_ = *t*_a_/*C*_L_ × ∑_*i*_*w*_*ij*_*I*_ph*,i*_ ∝ ∑_*i*_*w*_*ij*_*x*_*i*_, where *x*_*i*_ is the light intensity at *i*th photodiode, proportional to the photocurrent *I*_ph*,i*_ and *C*_L_ is the load capacitance of computing lines, which is determined by the number of connected photodiodes and parasitic capacitance between metal interconnects. To make it consistent, we connect one pair of positive capacitance compensation module (P-CCM) and negative capacitance compensation module (N-CCM) to computing lines V_+_ and V_−_, respectively (Fig. 2f, Extended Data Fig. 1d and Supplementary Note 2).

**Extended Data Fig. 2:**
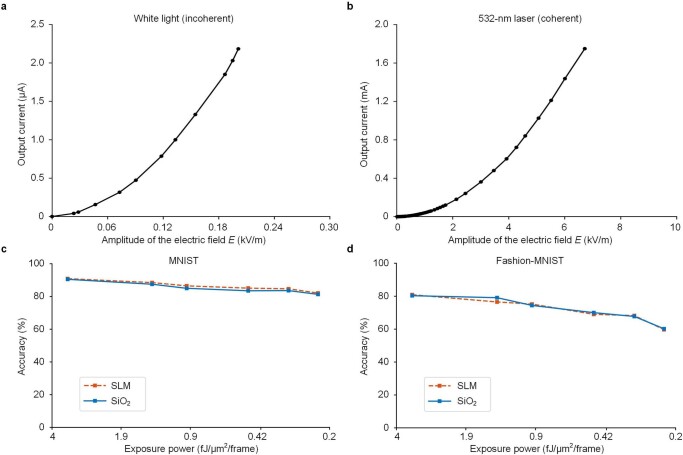
Measured nonlinearity in ACCEL and experimental results of ACCEL with SLM-based OAC and SiO_2_-based OAC. **a**,**b**, Experimentally measured nonlinear response of the photodiode (PD) in ACCEL versus different amplitudes of the electronic field for incoherent white light (**a**) and 532-nm coherent light (**b**). The area of the photosensitive surface *A* = 1.25 mm^2^; the speed of light *c* = 3.0 × 10^8 ^m/s; the dielectric constant *ϵ* = 8.9 × 10^−12 ^F/m; elementary charge *e* = 1.602 × 10^−19 ^C; Planck constant *h* = 6.626 × 10^−34 ^J ∙ s. **c**,**d**, Experimental classification accuracy of ACCEL with SLM-based OAC and SiO_2_-mask-based OAC under different exposure powers on the MNIST dataset (**c**) and the Fashion-MNIST dataset (**d**). The results are tested over the first 1,000 images in the testing dataset without selection. The pixel size of OAC is 9.2 µm and the diffraction distance is 150 mm here. Source Data

Then the whole computation process of ACCEL can be expressed as follows: **V**_**o**_ = *bf*(*w***x**), where **x** is the original input data; *w* is an equivalent multiplied matrix in OAC; *f*(*x*) is the nonlinear activation function generated with photodiodes (Extended Data Fig. 2 and Supplementary Note 3); *b* is the binary-weighted multiplied matrix in EAC; and **V**_**o**_ is the analog output pulse voltages. For the training of ACCEL, we model the complete analog physical process in OAC and EAC jointly and implement end-to-end fusion training (Methods).

## Performance characterization of ACCEL

**Fig. 3:**
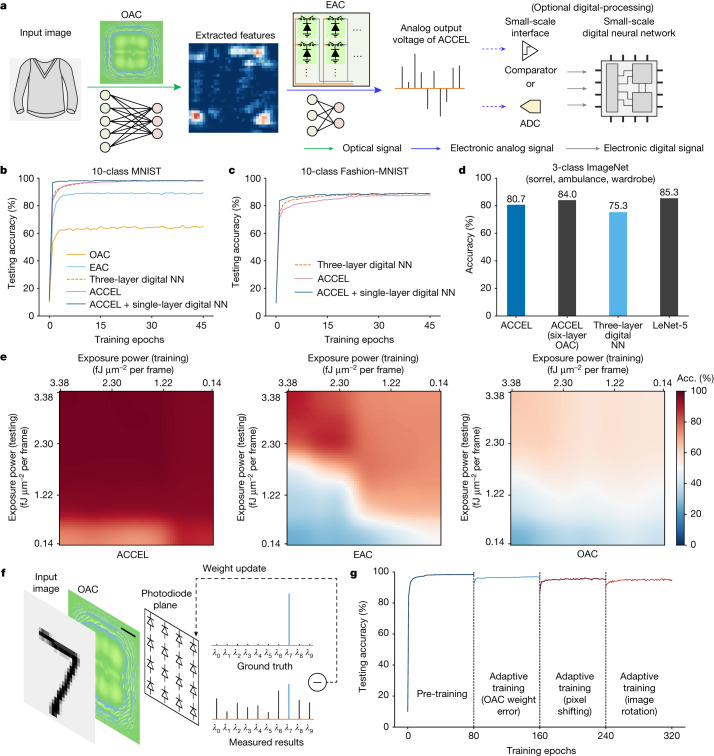
Numerical evaluation of ACCEL performance. **a**, The workflow of ACCEL for image classification. A large-scale OAC encodes the original inputs into small-scale features and the EAC computes the final results in an all-analog way. An optional small-scale digital NN can be connected to ACCEL for more complicated tasks or time-lapse applications at a low cost. **b–d**, Classification accuracies of different methods on 10-class MNIST (**b**), 10-class Fashion-MNIST (**c**) and 3-class ImageNet (**d**) obtained by numerical simulations. Detailed structures of all digital networks are listed in Supplementary Table 1. **e**, The map of classification accuracies of three different methods (ACCEL, EAC-only and OAC-only) trained under different incident light powers and tested under different incident light powers on the MNIST dataset obtained by numerical simulations. The light intensity is represented by the exposure energy in a 1-μm^2^ area for one image frame. **f**, Schematic of adaptive training, which fine-tunes the weights in EAC for the correction of system errors in practical applications. The output of OAC is captured by the photodiode array directly and is used to fine-tune the EAC weights with back propagation. Scale bar, 300 μm. The example input image is adapted from the MNIST dataset^40^ with permission. **g**, Testing accuracies of ACCEL with adaptive training to resist different kinds of manufacturing errors and misalignments obtained by numerical simulations. The pixel size of the phase mask in OAC is 3.0 µm, and the diffraction distance is 3 mm. For manufacturing error, the OAC weight is disturbed by Gaussian noises with zero mean value and a standard deviation of 0.26π. For misalignments, the 32 × 32 EAC input is shifted horizontally by one column and rotated clockwise by 5° around the centre. Source Data

A typical workflow of ACCEL is shown in Fig. 3a. The analog output voltages can be used directly as the classification results or sent into a small-scale digital NN with a single ADC or comparator to further improve the performance. Before the experimental demonstration, we first conducted numerical simulations to evaluate ACCEL quantitatively.

For the classification of 10-class handwritten digits on the MNIST dataset^40^, single-layer OAC-only and EAC-only can achieve classification accuracies of 66% and 89%, respectively (Fig. 3b), whereas the accuracy of ACCEL in the all-analog mode numerically reached 98%, competitive with a nonlinear three-layer digital fully connected NN (Supplementary Table 1). Even for a more challenging classification task (Fashion-MNIST of fashion products^41^), all-analog ACCEL numerically showed comparable performance to digital NNs (Fig. 3c). A small-scale fully connected digital layer (16 × 10 nodes) can also be connected to improve the accuracy to about 89% for Fashion-MNIST with negligible additional energy consumption and latency (Supplementary Note 4).

With a high-resolution mask in OAC for highly multiplexing of spatial modes, ACCEL can process more complicated high-resolution images (256 × 256 pixels), such as ImageNet^42^, which remains a challenge for state-of-the-art photonic processors (Fig. 3d). We compared ACCEL and digital NNs over a 3-class ImageNet classification task on sorrels, ambulances and wardrobes. All-analog ACCEL (with single-layer OAC) numerically achieved an accuracy of 80.7%, even better than a fully connected three-layer nonlinear digital NN (75.3%) with a large number of neurons (Supplementary Table 1). More diffractive layers in OAC further improve the performance. An all-analog ACCEL with a six-layer OAC numerically achieved an accuracy of 84.0%, comparable to a digital convolutional NN such as LeNet-5 (85.3%).

Another advantage of ACCEL is noise robustness. For practical applications in high-speed vision tasks, ultra-fast processing usually results in short exposure time. It leads to extremely low SNR because of shot noises, readout noises and electronic thermal noises, which may become the bottleneck for actual processing speed. ACCEL has intrinsic advantages in noise robustness^43^ by establishing a latent feature space to converge light together in local regions and reduced ADCs for lower readout noises when considering noises during training (Methods). Although testing accuracy on MNIST decreases with the reduction of light power, ACCEL trained with the consideration of noise slows down this process (Fig. 3e). Compared with individual OAC and individual EAC, ACCEL numerically shows better noise robustness. Even with extremely low-light intensity of 0.14 fJ μm^−2^ per frame, the testing accuracy of ACCEL remains high, which is important for high-speed vision tasks with both low-light-power input and strong readout noises in high-speed ADCs.

**Extended Data Fig. 3:**
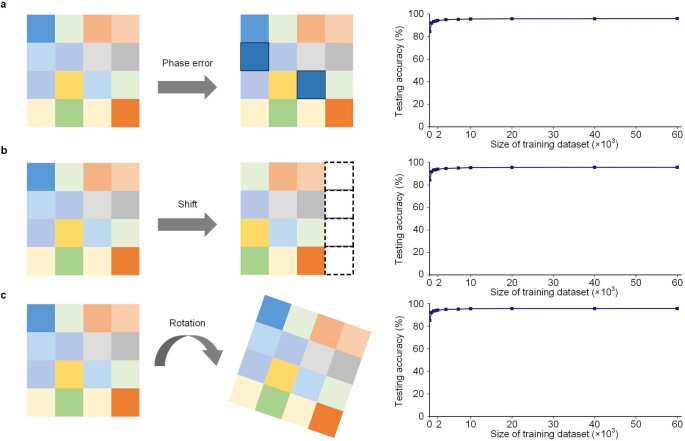
Evaluation of the robustness of adaptive training obtained by numerical simulations. **a**, The testing accuracy of ACCEL under the condition of fabrication errors in the OAC phase mask with adaptive training of different scales of training datasets. The phase pattern in OAC is disturbed by Gaussian noises with a mean value of zero and standard deviation of 0.26π to simulate the fabrication error. **b**, The testing accuracy of ACCEL under the condition of lateral misalignment between OAC and EAC with adaptive training of different scales of training datasets. The OAC and EAC are misaligned by shifting one column horizontally. **c**, The testing accuracy of ACCEL under the condition of rotation misalignment between OAC and EAC with adaptive training of different scales of training datasets. The OAC and EAC are misaligned by rotating clockwise by 5 degrees around the centre. All these results are tested on MNIST dataset. The scales of training dataset are 100, 500, 1,000, 1,500, 2,000, 4,000, 7,000, 10,000, 20,000, 40,000 and 60,000 images. To match the parameters in experiments, we set the pixel size of the phase mask in OAC as 9.2 µm and the diffraction distance as 150 mm here, and the neuron number in OAC is 264 × 264. Source Data

Another common bottleneck of analog computing is sensitivity to system errors induced by inevitable manufacturing defects and misalignment. Thus we establish an adaptive training method to fine-tune EAC with back propagation based on the intermediate OAC results captured by the photodiode array under its sensor mode (Fig. 3f). A small part of the training dataset (≤10%) can mitigate accuracy degradation due to phase errors of manufacturing or misalignment of shifting and rotation (Fig. 3g and Extended Data Fig. 3).

## High-performance image classification

**Fig. 4:**
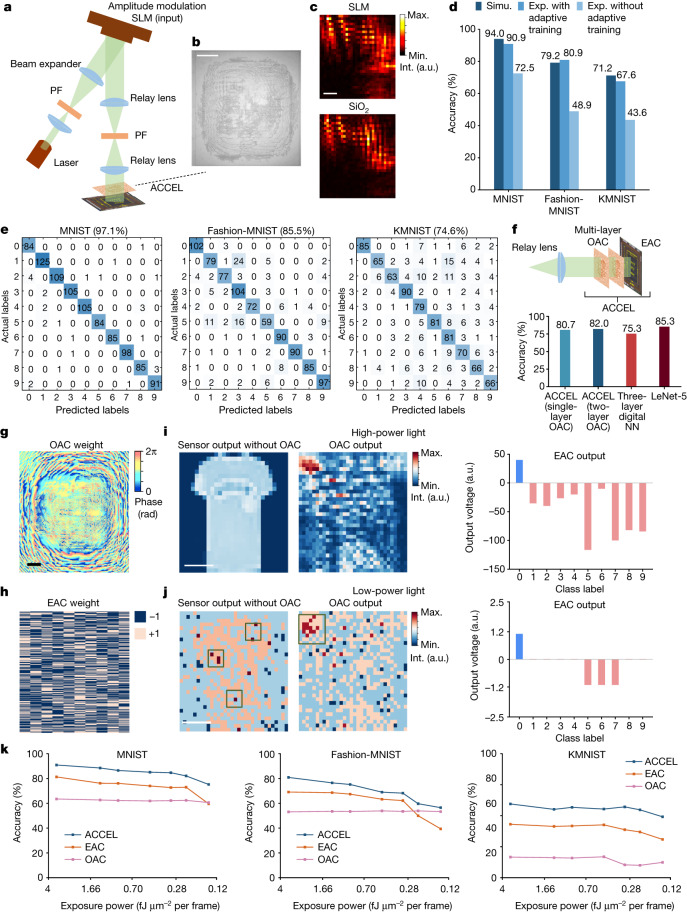
Experimental results of ACCEL for image classification. **a**, Experimental set-up to test ACCEL. PF, linear polarizer. The pixel size of the phase mask in OAC is 9.2 µm. **b**, Photograph of an etched eight-level phase mask with SiO_2_, serving as OAC. Scale bar, 500 μm. **c**, Experimental OAC output images obtained by a fixed SiO_2_ phase mask or a phase pattern generated by a phase-modulation SLM. Scale bar, 200 μm. **d**, Experimental classification accuracies of ACCEL with and without adaptive training on the MNIST, Fashion-MNIST and KMNIST datasets, compared with simulation accuracies. To match the parameters in experiments, we set the pixel size of the phase mask in OAC as 9.2 µm and the diffraction distance as 150 mm in the simulation. Simu., simulation; Exp., experiment. **e**, Confusion matrixes of ACCEL with single-layer small-scale digital NN (16 × 10 neurons) tested on the MNIST, Fashion-MNIST and KMNIST datasets. ACCEL and digital NN are connected through a 10-bit ADC and rectified linear unit nonlinearity is used between EAC and the digital NN. **f**, Experimental classification results of ACCEL with single-layer OAC and two-layer OAC on 3-class ImageNet classification, compared with digital fully connected and convolutional (LeNet-5) NNs (Supplementary Table 1). **g**,**h**, Experimental OAC weights (phase map) and EAC weights for the classification of Fashion-MNIST. Scale bar, 300 μm. **i**,**j**, Experimental example results for the Fashion-MNIST dataset with high-power light (5 fJ μm^−2^ per frame) (**i**) and low-power light (0.14 fJ μm^−2^ per frame) (**j**), including the direct output of the photodiode array with and without OAC and the output after both OAC and EAC. Scale bar, 300 μm. **k**, Experimental classification accuracies of ACCEL, OAC-only and EAC-only under different low-light conditions on the MNIST, Fashion-MNIST and KMNIST datasets. a.u., arbitrary unit; Max., maximum; Min., minimum; Int., intensity; Simu., simulation; Exp., experiment. Source Data

**Extended Data Fig. 4:**
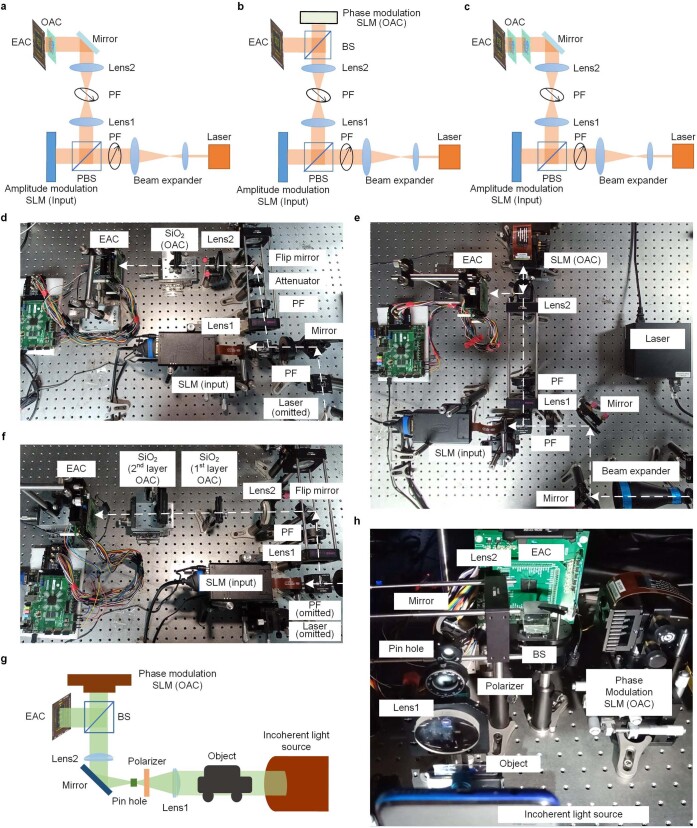
Experimental set-ups for testing ACCEL. **a–c**, Schematic of the experimental set-up for testing ACCEL with coherent light, using a fixed SiO_2_ phase mask as the single-layer OAC (**a**), SLM as the single-layer OAC (**b**), and two fixed SiO_2_ phase masks as the two-layer OAC (**c**). **d–f**, Photograph of the corresponding experimental set-up for testing ACCEL with coherent light, using a fixed SiO_2_ phase mask as the single-layer OAC (**d**), SLM as the single-layer OAC (**e**), and two fixed SiO_2_ phase masks as the two-layer OAC (**f**). **g**, Schematic of the experimental set-up for ACCEL computing with incoherent light. **h**, Photograph of the experimental system with incoherent light. The size of training and testing datasets are 400 and 100 sequences, respectively. Each sequence includes three frames. PF, linear polarizer; SLM, spatial light modulator; BS, beam splitter; PBS, polarization beam splitter.

To further verify the schematic of ACCEL, we conducted experiments with a fabricated ACCEL chip (Fig. 4a and Extended Data Fig. 4). We fabricated etched eight-level phase masks with SiO_2_ by overlay photolithography as OAC (Fig. 4b), integrated directly in front of the photodiode array in EAC. A phase-modulation spatial light modulator (SLM) can also be used as a reconfigurable diffractive phase mask in OAC, with similar experimental output as shown in Fig. 4c. Meanwhile, adaptive training in EAC can further reduce the influence of fabrication defects and alignment errors, leading to similar experimental classification accuracies of ACCEL with SiO_2_ phase mask and SLM under different exposure intensities (Extended Data Fig. 2c,d).

**Extended Data Fig. 5:**
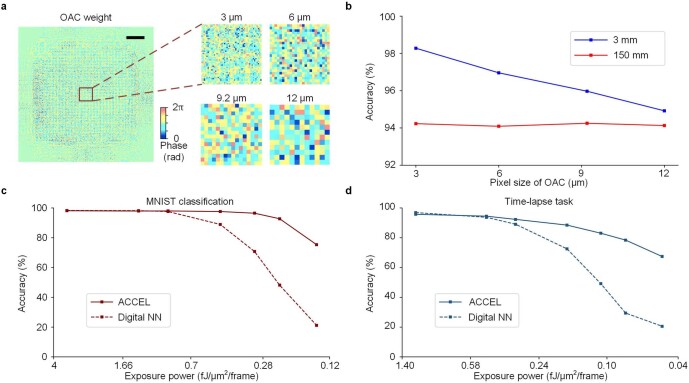
Numerical evaluation of ACCEL with different pixel sizes, diffractive distances in OAC, and various exposure power. **a**,**b**, Testing accuracies of ACCEL with different pixel sizes and diffractive distances on the MNIST dataset. Scale bar, 200 μm. The neuron number of OAC for the pixel sizes of 3 µm, 6 µm, 9.2 µm, and 12 µm are 500 × 500, 250 × 250, 164 × 164, and 126 × 126, respectively. **c**,**d**, The testing accuracy of ACCEL and digital NN under different exposure powers on MNIST and time-lapse tasks. ACCEL and digital NNs are trained under the exposure power of 3.38 fJ per µm^2^ per frame in (**c**) and 1.17 fJ per µm^2^ per frame in (**d**) (detailed network structures in Supplementary Table 1). We set the pixel size of OAC as 3 µm and the diffraction distance as 3 mm here. Source Data

We experimentally validated ACCEL over three datasets: handwritten digits (MNIST), fashion products (Fashion-MNIST) and cursive characters (KMNIST)^44^. To compare different tasks fairly, we used a phase-modulation SLM as the diffractive phase mask in OAC. ACCEL in all-analog mode experimentally achieved accuracies of 90.9%, 80.9% and 67.6% over MNIST, Fashion-MNIST and KMNIST, respectively, after adaptive training, which is close to the simulation performance (Fig. 4d). The decrease in simulation accuracy compared with Fig. 3 mainly results from the large pixel size of the SLM (9.2 µm), compared with the pixel size of 3 µm used in simulation (Extended Data Fig. 5a). Smaller pixel size can increase classification accuracy by enhancing diffraction effects with a shorter optimal diffraction distance between the mask and the sensor (Extended Data Fig. 5b). Furthermore, by connecting a small-scale digital NN (16 × 10 nodes) to ACCEL, experimental accuracies are enhanced to 97.1%, 85.5% and 74.6%, respectively, without sacrificing the systemic processing speed and energy consumption (Fig. 4e). To further show the advantage of ACCEL on more challenging tasks with high-resolution images, we used fabricated SiO_2_ phase masks in ACCEL to conduct 3-class ImageNet classification (Fig. 4f). Without connecting to any digital NNs, ACCEL experimentally achieved a testing accuracy of 80.7% with a single-layer OAC and EAC in an all-analog way. By increasing the layer number in OAC, experimental testing accuracy is further enhanced (82.0% for two-layer OAC), even higher than a three-layer, nonlinear, fully connected digital NN (75.3%) and comparable to a convolutional NN such as LeNet-5 (85.3%) (Supplementary Table 1).

Furthermore, we characterized the experimental accuracy on different datasets under different exposure powers (Fig. 4g–j). A reduction in light intensity by more than 35 times disrupts the image detected without OAC, which is also challenging for digital NN with similar scales (Extended Data Fig. 5c,d). However, OAC preserves the features well by integrating more photons in local regions, leading to better performance in low-light conditions on different tasks (Fig. 4k).

**Extended Data Fig. 6:**
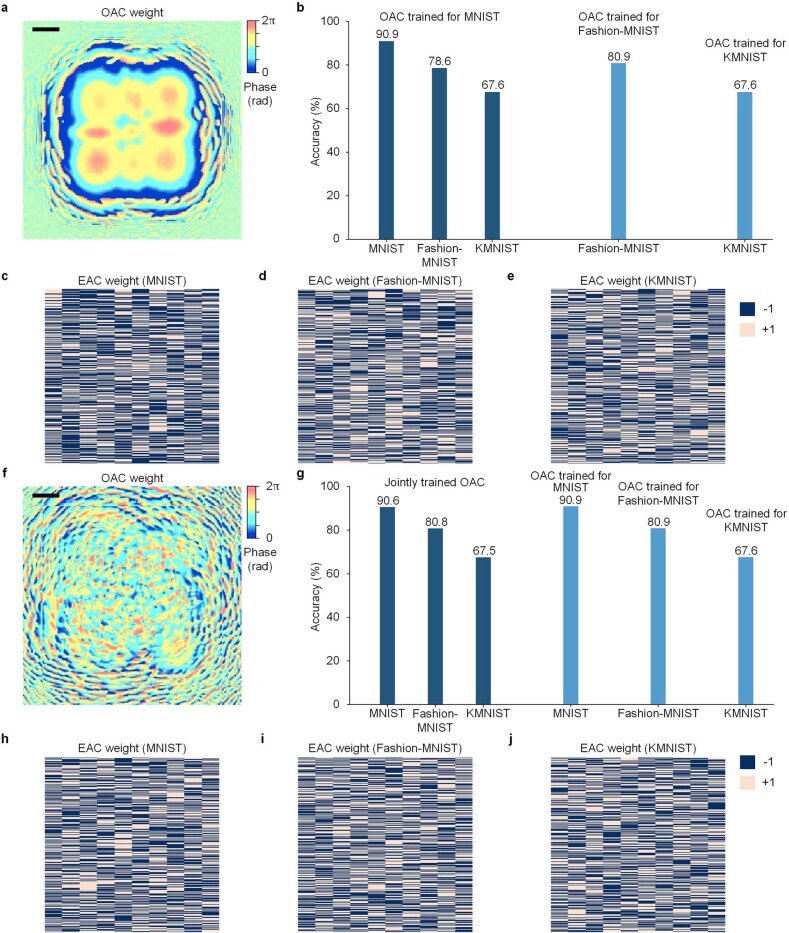
Experimental evaluation of the reconfigurability of all-analog ACCEL. **a**, OAC weights trained for the MNIST dataset. Scale bar, 300 μm. **b**, Experimental classification accuracies of ACCEL with the OAC mask trained for MNIST on the tasks of Fashion-MNIST and KMNIST. Fully reconfigured ACCEL trained for specific datasets are shown for comparisons. **c–e**, Reconfigured EAC weights for different datasets. **f**, The OAC weights jointly trained for MNIST, Fashion-MNIST and KMNIST datasets. Scale bar, 300 μm. **g**, Experimental classification accuracies of ACCEL with the jointly trained OAC mask on the tasks of MNIST, Fashion-MNIST and KMNIST. Fully reconfigured ACCEL trained for specific datasets are shown for comparisons. **h–j**, Reconfigured EAC weights for different datasets. The pixel size in OAC here is 9.2 μm.

Finally, we find that the partial reconfigurability of ACCEL in EAC enables ACCEL with the same fixed OAC to achieve comparable performance on different tasks to a fully reconfigured ACCEL in both EAC and OAC (Extended Data Fig. 6a–e). If we trained one OAC with all three datasets jointly, ACCEL with reconfigurable EAC for each dataset experimentally achieved even better generalization with only a slight accuracy loss (Extended Data Fig. 6f–j).

## High-speed time-lapse tasks

Apart from classification of static images, ACCEL facilitates high-speed processing of time-lapse tasks by providing a flexible and low-consumption interface from analog computing to digital memory and computing. With a simple digital chip connected, ACCEL can store serial outputs in memory and compute final results with a small-scale, single-layer network. Only a low-cost comparator instead of high-precision ADC can be used to convert analog signals into 1-bit digital signals, leading to much smaller energy consumption and latency (Extended Data Fig. 1e,f, Supplementary Note 5 and Supplementary Table 2).

**Fig. 5:**
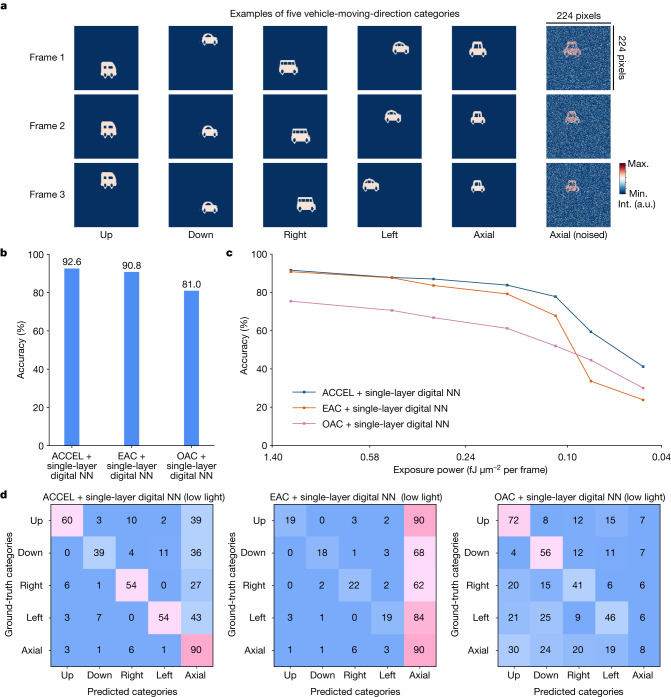
Experimental results of ACCEL for time-lapse tasks (video judgement). **a**, Illustrations of traffic dataset with a vehicle moving in five different directions. We present one example from each of the five moving-direction categories, and one noised example in low-light conditions from the axial category. The original position, speed and size of the vehicle are set randomly. **b**, Experimental accuracies of ACCEL connected with single-layer digital NN, EAC connected with single-layer digital NN and OAC connected with single-layer digital NN with the incident light condition of 5 fJ μm^−2^ per frame (Supplementary Table 1). We use the sign function between EAC and OAC and the digital NN as the nonlinear activation. The pixel size of the phase mask in OAC is 9.2 µm, and the diffraction distance is 150 mm. **c**, Experimental classification accuracies of ACCEL connected with single-layer digital NN, EAC connected with single-layer digital NN and OAC connected with single-layer digital NN under different incident light powers. **d**, Experimental confusion matrixes of ACCEL connected with single-layer digital NN, EAC connected with single-layer digital NN and OAC connected with single-layer digital NN under low-light condition (0.08 fJ μm^−2^ per frame). The EAC and OAC are connected to a single-layer digital NN (48 × 5 neurons) after conversion with 1-bit comparators, the same as ACCEL for fair comparisons. a.u., arbitrary unit; Max., maximum; Min., minimum; Int., intensity. Source Data

To show the potential applications in autonomous systems, we generated a traffic dataset, including 15 different vehicles to predict moving directions (Fig. 5a). Each sequence is composed of three 224 × 224 frames and can be divided into five categories: up, down, right, left and axial (Methods). We compared ACCEL with individual EAC and OAC on the video judgement task, each connected with a single-layer, fully connected digital NN. ACCEL experimentally achieved a prediction accuracy of 92.6% at 5.0 fJ μm^−2^ per frame, 1.8% and 11.6% higher than EAC and OAC, respectively (Fig. 5b). When reducing the light intensity for low-light conditions, we observed severe performance degradation in both EAC and OAC (Fig. 5c). By contrast, ACCEL experimentally maintained high accuracy, even better than a digital three-layer NN (Fig. 5c,d and Extended Data Fig. 5d).

## Computing speed and efficiency

**Fig. 6:**
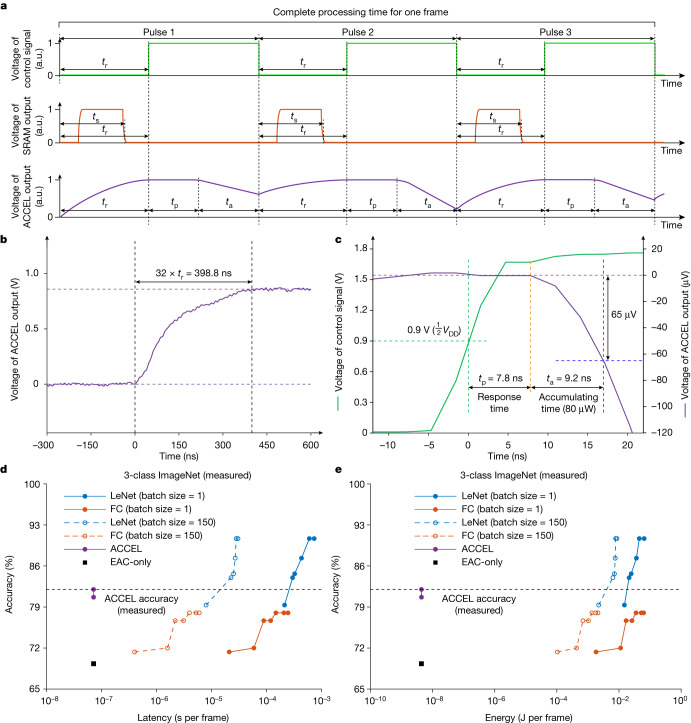
Experimental measurements of the processing time and energy consumption of ACCEL. **a**, Illustration of the voltage output of ACCEL (purple), SRAM (orange) and control signals (green) during the complete processing time of one frame. An example of 3-class classification is demonstrated. **b**, Experimentally measured average reset time of about 12.5 ns (*n* = 20,000). To avoid the influence of the buffer that disturbs the waveform, we measured the 32 times of reset time of about 398.8 ns. The steady-state voltage in the figure was about 0.86 V, which was the output voltage of the buffer when the input was 1.8 V (steady-state voltage of the computing line during reset operation). **c**, Measured average response time of 7.8 ns and average accumulating time of 9.2 ns when the incident light was 80 μW (*n* = 20,000). For better visualization, we added a 0.86 V offset in the voltage of ACCEL output. The position of the vertical green dashed line marks the start of the response time, when the control signal reaches half *V*_DD_. The position of the vertical orange dashed line marks the end of the response time when the output voltage starts to drop. The vertical blue dashed line marks the end of the accumulating time when the drop of output signal has enough contrast to be distinguished. The accumulating time varies with the incident light power. **d**,**e**, Experimentally measured curves of classification accuracies on 3-class ImageNet versus measured systemic computing latency (**d**) and energy consumption (**e**) of one frame for comparisons among ACCEL, digital fully connected (FC) NN and convolutional NN (LeNet) with different layer numbers or batch sizes implemented on NVIDIA A100. FC, fully connected. Detailed network structures are listed in Supplementary Tables 1, 6 and 7. a.u., arbitrary unit. Source Data

**Extended Data Fig. 7:**
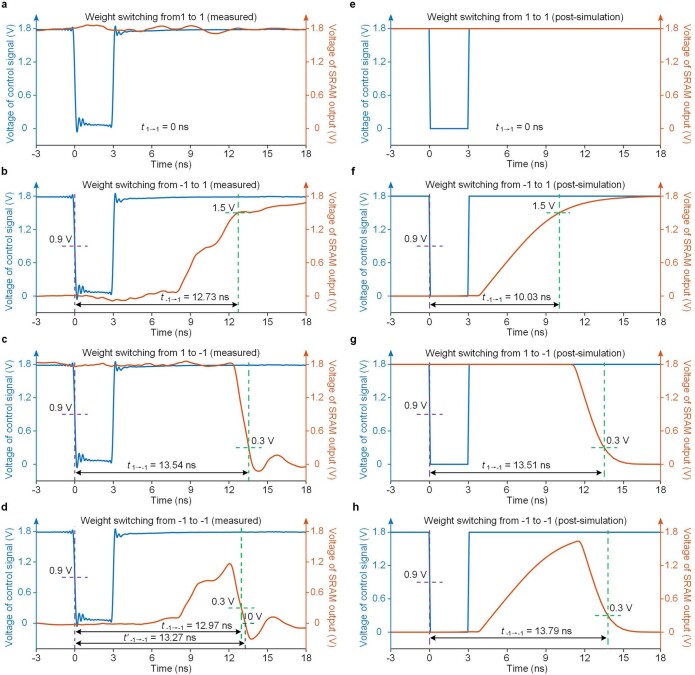
Experimentally measured latency for weight switching by SRAM in ACCEL. **a–d**, Experimentally measured time of weight switching of SRAM in all four situations: from 1 to 1 (**a**), from −1 to 1 (**b**), from 1 to −1 (**c**) and from −1 to −1 (**d**). **e–h**, Calculated time of weight switching by SRAM with parameters from the foundry with post-simulation in all four situations: from 1 to 1 (**e**), from −1 to 1 (**f**), from 1 to −1 (**g**) and from −1 to −1 (**h**). Source Data

As shown in Fig. 6a, the complete processing time of ACCEL for each frame is composed of three parts: (1) reset time *t*_r_, used to pre-charge computing lines with a uniform voltage and avoid residual effects of previous pulses; (2) response time *t*_p_, including complete propagation time for both OAC and EAC from analog light signals to analog electronic outputs; and (3) accumulating time *t*_a_, for the output signal to accumulate voltages distinct enough above the systemic noise threshold. The SRAM latency *t*_s_ for weight update in EAC for each pulse is completed within the reset time (Fig. 6a, orange line) and, therefore, does not contribute to complete processing time experimentally (Extended Data Fig. 7 and Supplementary Note 6).

**Extended Data Fig. 8:**
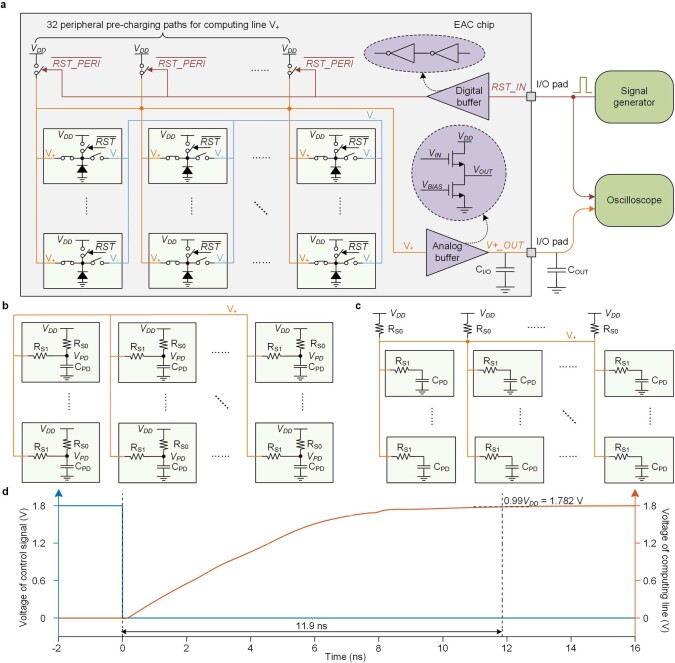
Measurement of reset time, response time and accumulating time of ACCEL and circuit modelling of reset operation. **a**, Configurations of reset operation with the voltage-readout signal chains for computing lines. The voltages of computing lines are read out with an on-chip buffer for observations. The signal generator provides the control signal, which enables the reset operation when the voltage of control signal is low (ground voltage), and enables the computing process when the voltage of control signal is high (supply voltage). **b**, Circuit modelling of the pre-charging process of the computing line with local charging paths. **c**, Circuit modelling of the pre-charging process of the computing line with peripheral charging paths. **d**, Post-simulated timing diagram of the reset operation of computing line with local pre-charging paths. Source Data

We established two experiments to measure the reset time, response time and accumulating time separately (Methods and Supplementary Notes 7 and 8). As the reset time is an intermediate process, the direct measurement may be distorted because of the limited output bandwidth in the chip. We specifically extended *t*_r_ by 32 times and measured the upper limit of 32*t*_r_ in the chip (Methods and Extended Data Fig. 8), which is about 398.8 ns (Fig. 6b). Therefore, the experimental upper limit of reset time *t*_r_ is 12.5 ns, which agrees well with the post-simulation results with Cadence (Extended Data Fig. 8d and Supplementary Note 7). Because the noise variance of the output in EAC is 6.43 μV according to the chip characteristic (Supplementary Note 8), we set the threshold of voltage drop as 65 μV in ACCEL. The measured average response time is 7.8 ns and the average accumulating time is 9.2 ns when the incident light is 80 μW (Fig. 6c). The accumulating time decreases with the increase of exposure intensity as measured in Supplementary Table 3, leading to the maximum of 2.1 ns for the incident light of 350 μW. We here used a clock frequency of 500 MHz (2 ns as a single clock period in ACCEL). When the incident light is 0.14 fJ μm^−2^ per frame (3.5 mW), we used 12 clock periods for one pulse, allowing adequate time for correct operation. Therefore, the experimental processing time of ACCEL for one pulse is 24 ns, and the complete processing time of ACCEL including three pulses for 3-class classifications is about 72 ns. Our fabricated ACCEL for 3-class ImageNet classification contains two 400 × 400 SiO_2_ OAC layers and a 1,024 × 3 EAC layer, leading to a minimum number of operations per frame as 3.28 × 10^8^ (Supplementary Note 9). Consequently, the measured computing speeds of ACCEL at the system level for 3-class ImageNet is about 4.55 × 10^3^ TOPS (Supplementary Note 9 and Supplementary Table 4).

The measured average systemic energy consumption of ACCEL for 3-class ImageNet classification is 4.4 nJ, composed of energy consumption from the laser, SRAM, control unit and EAC computing (Methods). Hence the experimental systemic energy efficiency of ACCEL for 3-class ImageNet is 7.48 × 10^4^ TOPS W^−1^ (74.8 peta-OPS W^−1^). Detailed calculations are listed in Supplementary Notes 4 and 9 and Supplementary Tables 4 and 5.

**Extended Data Fig. 9:**
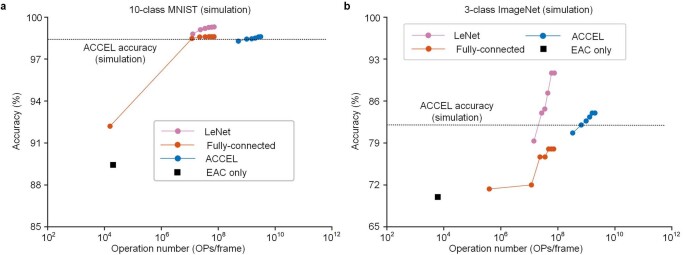
Comparisons of the testing accuracies among ACCEL and digital electronic neural networks with different operation numbers over MNIST and 3-class ImageNet classification, obtained by numerical simulations. **a**, 10-class classification accuracies on MNIST of ACCEL, digital electronic fully-connected NN and convolutional NN (LeNet) with different layer numbers (corresponding to different operation numbers) obtained by numerical simulation. **b**, 3-class classification accuracies on ImageNet of ACCEL, digital electronic fully-connected NN and convolutional NN (LeNet) with different layer numbers (corresponding to different operation numbers) obtained by numerical simulation. Detailed structures of the digital NNs are listed in Supplementary Tables 6, 7. Source Data

For practical applications, task performances also vary with different network structures, such as fully connected, convolutional or diffractive networks. Therefore, we proposed a new metric, termed as LeNet-equivalent operation number, to evaluate the effective operation number of ACCEL for fair comparisons with digital NNs. The LeNet-equivalent operation number equals the operation number of LeNet to reach the same accuracy as ACCEL on a complicated task before performance saturation, based on the fact that more operation numbers increase task performances for a specific network structure. When achieving 82.0% on 3-class ImageNet classification, the LeNet-equivalent operation number of ACCEL is 2.17 × 10^7^ (Extended Data Fig. 9). Therefore, the experimental systemic LeNet-equivalent computing speed and energy efficiency of ACCEL are 301.39 TOPS and 4.95 × 10^3^ TOPS W^−1^, respectively, remaining much higher than state-of-the-art digital and photonic devices (Extended Data Table 1).

Finally, we provided a direct validation by measuring end-to-end latency and energy consumption of ACCEL and different kinds of digital NNs implemented on state-of-the-art GPU for the same task (Supplementary Tables 6 and 7). When processing images in serial with the same test accuracy, ACCEL experimentally achieved a computing latency of 72 ns per frame and energy consumption of 4.38 nJ per frame, much smaller than NVIDIA A100 whose latency and energy consumption are about 0.26 ms per frame and 18.5 mJ per frame, respectively (Fig. 6d,e). Regardless of either way to calculate the operation number, all-analog ACCEL experimentally reduces the systemic latency and energy consumption by orders of magnitude compared to digital NNs on state-of-the-art GPU (NVIDIA A100) when achieving the same accuracy in practical applications.

## Discussion

### Scalability of ACCEL

The performance of ACCEL can be further improved if we add more layers to OAC^45^ or re-design EAC for parallel outputs with more sensitive photodiode arrays. Increasing bits stored in SRAM can extend the maximum number of classification categories of ACCEL. In the aspect of manufacturing costs, we now only used standard 180-nm complementary metal-oxide-semiconductor (CMOS) technology for EAC and low-cost SiO_2_-etched panels for OAC, whereas state-of-the-art GPUs and tensor processing units require much more advanced CMOS processes. Advanced CMOS technology can be used in ACCEL to massively reduce the power consumption of the control unit operating at a higher clock frequency.

Moreover, more complicated network structures in OAC and EAC can also be implemented in an all-analog way for more challenging tasks, as verified by our previous works^46,47^. Neural networks with a larger size can be implemented in the EAC part for complicated nonlinear processes. With the low-power consumption and low latency in optoelectronic conversion, several ACCELs can be cascaded in the future to implement a large-scale DNN by using the whole ACCEL as an encoder with a very small size of output nodes, and a digital micro-mirror device and light source to convert these nodes again from electronic signals to optical signals^6^. The EAC reconfigurability and the proposed adaptive training allow cascaded ACCEL to eliminate severe error accumulations.

Optical computing has native advantages in vision tasks as the passive light from the environment carries the information itself. However, existing ONNs usually require coherent light sources and are hard to apply in passive detection, which notably reduces the computing speed during light–light conversion. With strong noise robustness in low-light conditions, ACCEL can be directly used in processing incoherent or partially coherent light fields as long as we reduce the aperture of the detection imaging system to enhance the spatial coherence. For verification, we conducted an experiment on video judgement by illuminating the object with the flashlight on a cell phone (Extended Data Fig. 4g,h and Supplementary Video 1). High-speed recognition was obtained with an experimental classification accuracy of 85% over 100 testing samples, indicating the capability of ACCEL to compute with incoherent light directly. This capability can not only further reduce the power consumption but also improve the processing speed in practical applications without requirement of extra sensors and light sources to capture and reproduce the scene.

By combining the advantages of both photonic and electronic computing, ACCEL achieves a systemic computing speed of 4.55 × 10^3^ TOPS and an energy efficiency of 7.48 × 10^4^ TOPS W^−1^ experimentally, orders of magnitude higher than state-of-the-art methods, and maintains competitive accuracy in diverse intelligent vision tasks, compared with digital NNs in electronic processors. Besides serving as a general smooth interface from analog optical signals to digital signals, ACCEL also opens up a new horizon for broad practical applications of optoelectronic analog computing such as wearable devices, robotics, autonomous driving, industrial inspections and medical diagnosis.

## Methods

### Experimental set-up and materials

Sketches and experimental set-ups of ACCEL both with SLM and fixed SiO_2_ as single-layer OAC are shown in Extended Data Fig. 4. The diffractive distances of the SLM and the SiO_2_ mask for single-layer OAC are both set as 150 mm. The diffractive distances of ACCEL with two-layer OAC are set as 140 mm between the layers of OAC and 145 mm between the OAC and EAC. For coherent-light experiments, we used a single-mode 532-nm laser (Changchun New Industries Optoelectronics Tech, MGL-III-532-200mW). The laser is first collimated with the beam expander and illuminates the amplitude-modulation-only SLM (HOLOEYE Photonics, HES6001), which is used to input images and videos with linear polarizers and a polarized beam splitter. The testing data is the first 1,000 images from the original testing dataset without selection in MNIST, Fashion-MNIST and KMNIST classification experiments and first 500 sequences from the original testing dataset without selection in time-lapse experiments. For the partial-coherent-light experiment, we used a flashlight on a cell phone as the light source and a 4f relay system as the imaging system to relay the light field to ACCEL.

We used phase-modulation-only SLM (Meadowlark Optics, P1920-400-800-PCIE) or SiO_2_ plates as OAC in ACCEL. By overlay photolithography, the depth level of the SiO_2_ phase mask is 3 bits with a maximum etch depth of 1,050 nm and minimum line width of 9.2 μm. The thickness of the plate is 0.6 mm and the material is jgs1. The analog electronic chip for EAC is fabricated with the 180-nm standard CMOS process of the Semiconductor Manufacturing International Corporation. The supply voltage is 1.0 V for the on-chip controller but 1.8 V for other modules of EAC. The chip area is about 2.288 mm × 2.045 mm. The photodiode array has a resolution of 32 × 32 with a pixel size of 35 μm × 35 μm and a fill factor of 9.14%.

### Weight storage in EAC

As shown in Fig. 2h, an SRAM macro is used in each pixel to store binary weights, which controls the switches S2 and S3 to connect the photodiode to computing line V_+_ or V_−_. The SRAM macro is composed of 16 SRAM units, so that computation of binary fully connected networks supports up to 16 output nodes (Extended Data Fig. 1a). Multiple outputs of the binary fully connected network are calculated serially along time (Fig. 1b). To compute the value of a new output, the corresponding weight in the SRAM macro is first read out to control the switches S2 and S3, and the photocurrent accumulation process sequentially begins. The standard eight-transistor SRAM structure, which adopts a separate write-word-line and a separate read-word-line for the write operation and read operation, is used for SRAM circuit implementation (Extended Data Fig. 1b).

### Operation pipeline of EAC

Before the calculation by each pulse, switch S1 in each pixel (Fig. 2h) is first turned on to reset the voltage of the computing lines V_+_ and V_−_ to the same supply voltage *V*_DD_, to avoid the residual effect of previous pulses. During this reset time, the SRAM macro updates the switch to connect either S2 or S3 based on the weight *w*_*ij*_ for the *j*th output pulse. The weights *w*_*ij*_ for each output node are then sequentially read out from the SRAM macro during each pulse to control the switches S2 and S3, leading to *N*_output_ output pulses of the fully connected neural network implemented sequentially in the temporal domain. Finally, a comparator is used to find the maximum output voltage, which corresponds to the classification result in the all-analog mode. The timing diagram of each signal in EAC during calculation is shown in Extended Data Fig. 1c.

### Training of ACCEL

For the training of ACCEL, we model the complete analog physical process in both OAC and EAC jointly with Tensorflow, including the modulation and light diffraction in OAC, the nonlinearity using photoelectronic conversion and the equivalent matrix multiplication in EAC. We implemented end-to-end fusion training by stochastic gradient descent and back propagation with the loss function as: *l* = *C*(*S*(**V**_**o**_), **G**), where *C*(*x*) is the function of cross entropy; *S*(*x*) is the function of softmax; **G** is the vector of correct labels and **V**_**o**_ is the output results—that is, analog output voltages of ACCEL. After training, we obtained both the phase masks in OAC and the weights *w*_*ij*_ in EAC.

### Modelling of low-light conditions

In addition to the intrinsic shot noise of the light modelled with a Poisson distribution, noises such as the thermal noises in EAC and the readout noises after EAC become relatively dominating when the input light intensity reduces either by reducing the input laser power or reducing the exposure time. For simplification, we modelled the comprehensive influences of the two kinds of noises as two random Gaussian variations on OAC and EAC outputs, respectively. The mean values of the Gaussian distributions were set as zero and the variances were set as constants. We multiply the normalized OAC output with a coefficient corresponding to the change in the light intensity. The variance of the OAC output noise *σ*_OAC_ was calibrated with the mean SNR of experimental OAC outputs. The variance of the EAC output noise *σ*_EAC_ was computed with the mean SNR of experimental EAC outputs. The numerical simulations accord well with the experimental results (Figs. 3e and  4k).

### Measurement of the reset time

Each pixel unit contains a local reset switch controlled by the RST signal to connect the photodiode to the power supply *V*_DD_ (Extended Data Fig. 8a). When the reset switch is turned on to enable the reset operation for the computing line, the photodiodes are charged to supply voltage *V*_DD_ with the local charging paths in each pixel. The charging speed is determined by the RC time constant *τ* = *R*_S0_*C*_PD_, where *C*_PD_ is the capacitance of the photodiode and *R*_S0_ is the on-resistance of the reset switch (Extended Data Fig. 8b). The transient function of the voltage of the photodiode with time can be formulized with the standard RC charging function as *V*_PD_(*t*) = *V*_DD_ – (*V*_DD_ – *V*_0_)e^−*t*/*τ*^, where *V*_0_ is the initial voltage of the photodiode. Theoretically, *V*_PD_ approaches the stable-state-voltage *V*_DD_ as time *t* approaches infinite. Here, we consider *V*_PD_ reaching the stable state when the increase of *V*_PD_ from *V*_0_ is larger than 99% of *V*_DD_ – *V*_0_, and thus the reset time is derived as *t*_r_ = 4.6*τ*, which is about 12 ns according to the post-simulation result (Extended Data Fig. 8d). The voltage of the computing line is read out with an on-chip buffer to the chip I/O pin and recorded by an oscilloscope. However, because of the limited bandwidth of the on-chip buffer, the output signal may be distorted when the computing line is charged at a high speed, affecting the precision of the measured reset time. To measure the reset time more precisely, we used peripheral charging paths instead of the in-pixel local charging paths for the reset operation. The 1,024 photodiodes in the pixel array were all connected to the computing line V_+_, and V_+_ was connected to the power supply *V*_DD_ with 32 peripheral switches (Extended Data Fig. 8a,c). Thus, the RC time constant of the peripheral charging path becomes *τ*′ = (*R*_S0_/32) × (1,024 × *C*_PD_) = 32*τ*, resulting in the reset time of about 32 times 12 ns. The experimentally measured reset time with peripheral charging paths is presented in Fig. 6b. The horizontal dashed lines are the average values of the steady-state voltage. The vertical dashed lines are the intersection points of the signal with the steady-state voltages (horizontal lines). Furthermore, if we consider the charging resistance introduced by *R*_S1_, the reset time with peripheral charging paths is larger than 32 times that with local charging paths. Therefore, the time of dividing the measured 398.8 ns in Fig. 6b by 32—that is, 12.5 ns is the upper limit of the experimental reset time, according well with the post-simulation results with Cadence (Extended Data Fig. 8 and Supplementary Note 7).

### Measurement of systemic computing speed

We implemented experiments to measure the three parts of the complete processing time of ACCEL (Fig. 6b,c). As mentioned before, the experimentally measured upper limit of the single-pulse reset time *t*_r_ is 12.5 ns. The measurements of the remaining response time and accumulating time are displayed in Fig. 6c. The beginning of the response time is the time when the control signal (green line) reaches half *V*_DD_ (0.9 V here), indicating the state of the reset switch in each pixel beginning to flip. The end of the response time is the time when the signal starts to drop, which is also the beginning of the accumulating time (orange line). The end of the accumulating time is the time when the output voltage drops to a certain level with enough SNR to distinguish (blue line). Because the noise variance of the output in our EAC is about 6.43 μV according to the characteristic of the chip (Supplementary Note 8), we set the threshold of voltage drop as 65 μV (more than 20 dB) in ACCEL. Input light with higher power will increase the descent rate of the output voltage, leading to further reduction of the accumulating time at the cost of larger power consumption, whereas the response time is rather similar under different light powers. The experimentally measured response time is about 7.8 ns, and the measured accumulating time is 9.2 ns when the incident light is 80 μW. Therefore, the response time and accumulating time are together 17.0 ns for an incident light of 80 μW. Moreover, we experimentally measured the accumulating time for the output voltage to reach 20 dB under different light powers in Supplementary Table 3. When the incident light is above 350 μW, the accumulating time is within 2.1 ns according to measurement.

The switch between reset and response requires the control signal from the control unit. A high-frequency clock precisely matching the processing time can increase the processing speed at the cost of high power consumption. Although the power of the control units increases along with the clock frequency, it also results in higher computing speed. We here used a clock frequency of 500 MHz with 2 ns for a single clock period in ACCEL. When the incident light equals or is above 0.14 fJ μm^−2^ per frame (3.5 mW), we used 12 clock periods for the reset, response and accumulating time, allowing adequate time for correct operation in each procedure. Therefore, the experimental complete processing time of ACCEL for one pulse is about 24 ns. Because the number of pulses for one frame in ACCEL depends on the number of classification classes, the complete processing time of ACCEL, including three pulses for 3-class classifications and 10 pulses for 10-class classifications, is about 72 ns and 240 ns, respectively. Our fabricated ACCEL for 3-class ImageNet classification contains two 400 × 400 SiO_2_ OAC layers and a 1,024 × 3 EAC layer. Our fabricated ACCEL for 10-class MNIST classification contains a 264 × 264 OAC layer and a 1,024 × 10 EAC layer. Therefore, they have a minimum number of operations per frame as 3.28 × 10^8^ and 1.43 × 10^8^ for 3-class ImageNet and 10-class MNIST classification, respectively (detailed calculations in Supplementary Note 9 and Supplementary Table 4). As a result, the experimental computing speeds of ACCEL at the system level for 3-class ImageNet and 10-class MNIST classifications are about 4.55 × 10^3^ TOPS and 5.95 × 10^2^ TOPS, respectively.

### Measurement of systemic energy efficiency

Because OAC implemented with fixed SiO_2_ phase masks is passive, the energy consumption only contains the incident light energy and all the energy for the electronic devices in ACCEL, including the energy for pre-charging and computing with photocurrents in EAC, the energy used to store, read and switch weights in SRAM and the energy of the control unit to switch ACCEL between pre-charging and computing.

For the 10-class MNIST classification under the incident light energy of 0.14 fJ μm^−2^ per frame, the measured energy of light (laser energy instead of the energy arriving at ACCEL) is about 11.8 nJ for the processing duration. The energy consumption of SRAM and the control unit for one frame are experimentally measured as 1.2 nJ and 2.0 nJ, respectively. The energy consumption of EAC computing is about 38.5 pJ. Therefore, the systemic energy consumption of the ACCEL at 0.14 fJ μm^−2^ per frame for 10-class MNIST classification is 15.0 nJ. For 3-class ImageNet classification when achieving the classification accuracy of 82.0% experimentally, the measured energy consumption of laser, SRAM, control unit and EAC computing for one frame are about 3.4 nJ, 0.4 nJ, 0.6 nJ and 11.6 pJ, respectively. The systemic energy consumption of ACCEL for 3-class ImageNet classification is 4.4 nJ. We also listed these detailed numbers and calculations in Supplementary Note 9 and Supplementary Table 4.

As a result, the experimental systemic energy efficiency of ACCEL for 10-class MNIST and 3-class ImageNet are 9.49 × 10^3^ TOPS W^−1^ and 7.48 × 10^4^ TOPS W^−1^ (74.8 peta-OPS W^−1^), respectively. Similarly, the systemic energy efficiency of ACCEL connected with a small-scale digital layer for 10-class MNIST and time-lapse tasks are 5.88 × 10^3^ TOPS W^−1^ and 4.22 × 10^3^ TOPS W^−1^, respectively (detailed calculations are listed in Supplementary Notes 4 and 9 and Supplementary Tables 4 and 5).

### End-to-end comparison between ACCEL and state-of-the-art GPU

We provided a direct validation by measuring end-to-end latency and energy consumption of ACCEL and different kinds of digital NNs implemented on state-of-the-art GPU when experimentally achieving the same accuracy on the same task. Because MNIST is a relatively simple vision task, leading to saturation of the classification accuracy (Extended Data Fig. 9a and Supplementary Table 6), we used a more complicated vision task for testing (3-class ImageNet classification), which has a higher resolution (256 × 256 pixels here) and much more details than MNIST (Extended Data Fig. 9b and Supplementary Table 7). For state-of-the-art GPU, we used NVIDIA A100, whose claimed computing speed reaches 156 TFLOPS for float32 (ref. ^33^). ACCEL with two-layer OAC (400 × 400 neurons in each OAC layer) and one-layer EAC (1,024 × 3 neurons) experimentally achieved a testing accuracy of 82.0% (horizontal dashed line in Fig. 6d,e). Because OAC computes in a passive way, ACCEL with two-layer OAC improves the accuracy over ACCEL with one-layer OAC at almost no increase in latency and energy consumption (Fig. 6d,e, purple dots). However, in a real-time vision task such as automatic driving on the road, we cannot capture multiple sequential images in advance for a GPU to make full use of its computing speed by processing multiple streams simultaneously^48^ (examples as dashed lines in Fig. 6d,e). To process sequential images in serial at the same accuracy, ACCEL experimentally achieved a computing latency of 72 ns per frame and an energy consumption of 4.38 nJ per frame, whereas NVIDIA A100 achieved a latency of 0.26 ms per frame and an energy consumption of 18.5 mJ per frame (Fig. 6d,e).

### Benchmarking against digital NNs

Detailed structures of digital NNs used to compare with ACCEL are all listed in Supplementary Table 1.

### Dataset availability for video judgement in traffic scenes

The full version of our video dataset with five categories for moving-direction prediction in traffic scenes can be accessed at GitHub (https://github.com/ytchen17/ACCEL/tree/v1.0.1/video%20judgment%20dataset). It is composed of 10,000 different sequences with 8,000 for training and 2,000 for testing. The types, initial positions, moving speeds and sizes of the vehicles are all set randomly in the dataset for generalization.

## Online content

Any methods, additional references, Nature Portfolio reporting summaries, source data, extended data, supplementary information, acknowledgements, peer review information; details of author contributions and competing interests; and statements of data and code availability are available at 10.1038/s41586-023-06558-8.

### Supplementary information

Supplementary Information The Supplementary Information file contains Supplementary Notes 1–9 and Supplementary Tables 1–7.

Supplementary Video 1 Experiment of ACCEL with incoherent light. The flashlight on a cell phone is used as an incoherent light source to illuminate the object (a pattern of a vehicle) moving along a specific direction. ACCEL is trained for a time-lapse task to classify the five moving directions of the object (up, down, left, right and axial) over 400 training sequences. ACCEL achieved all correct results over the demonstrated five examples in different categories.

### Source data

Source Data Fig. 2

Source Data Fig. 3

Source Data Fig. 4

Source Data Fig. 5

Source Data Fig. 6

Source Data Extended Data Fig. 2

Source Data Extended Data Fig. 3

Source Data Extended Data Fig. 5

Source Data Extended Data Fig. 7

Source Data Extended Data Fig. 8

Source Data Extended Data Fig. 9

## Figures and Tables

**Extended Data Table 1:**
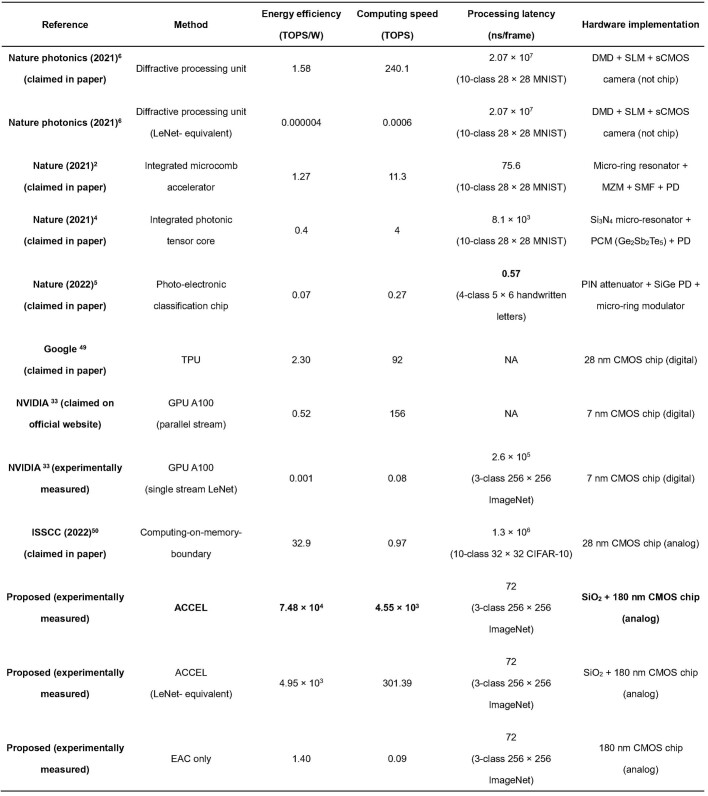
Main experimental performance of ACCEL compared with state-of-the-art electronic and photonic devices

We use the energy efficiency, computing speed, and processing latency at the system level here for comparisons^49,50^. DMD, digital micro-mirror device; SLM, spatial light modulator; sCMOS, scientific complementary metal oxide semiconductor; MZM, Mach-Zehnder modulator; SMF, single-mode fibre; PD, photodetector; PCM, phase-change materials; PIN, P-doped-intrinsic-N-doped; CMOS, complementary metal oxide semiconductor. For the performance of GPU, we listed both the claimed computing speed and efficiency from the official website (parallel stream for processing multiple input images simultaneously) and our experimentally measured results on the same task as ACCEL (single stream for single input image in real-time processing) for fair comparisons.

## Data Availability

The data supporting the findings of this study are available in the main text, Extended Data, Supplementary Information, source data and Zenodo (10.5281/zenodo.8174034). Source data are provided with this paper.

## Code availability

The algorithms and codes supporting the findings of this study are available in the main text, Extended Data and GitHub (https://github.com/ytchen17/ACCEL).
