# Supplementary material for: All-analog photoelectronic chip for high-speed vision tasks
Source: Nature. 2023 Oct 25;623(7985):48–57. doi: 10.1038/s41586-023-06558-8 (PMC10620079; doi:10.1038/s41586-023-06558-8)
Supplement: Supplementary file 1 — The Supplementary Information file contains Supplementary Notes 1–9 and Supplementary Tables 1–7. [file 41586_2023_6558_MOESM1_ESM.pdf]

nature portfolio

https://doi.org/10.1038/s41586-023-06558-8

Supplementary information

All-analog photoelectronic chip for high-speed vision tasks

In the format provided by the authors and unedited

Supplementary Information for

All-analog photo-electronic chip for high-speed vision tasks

Yitong Chen[1*], Maimaiti Nazhamaiti[2*], Han Xu[2*], Yao Meng[3], Tiankuang Zhou[1,3,4], Guangpu Li[1,3], Jingtao Fan[3], Qi Wei[6], Jiamin Wu[1,3†], Fei Qiao[2†], Lu Fang[2,3,5†], Qionghai Dai[1,3,5†]

[1]*Department of Automation, Tsinghua University, Beijing, 100084, China*

[2]*Department of Electronic Engineering, Tsinghua University, Beijing, 100084, China*

[3]*Institute for Brain and Cognitive Sciences, Tsinghua University, Beijing, 100084, China*

[4]*Shenzhen International Graduate School, Tsinghua University, Shenzhen, 518071, China*

[5]*Beijing National Research Center for Information Science and Technology, Tsinghua University, Beijing 100084, China*

[6]*Department of Precision Instruments, Tsinghua University, Beijing, 100084, China*

[*]*These authors contributed equally to this work*

[†]Correspondence: qhdai@tsinghua.edu.cn (Q. D.), fanglu@tsinghua.edu.cn (L. F.), qiaofei@tsinghua.edu.cn (F. Q.), wujiamin@tsinghua.edu.cn (J. W.)

Table of Contents

**Supplementary Note 1**	The operation principle of ACCEL SRAM unit.
**Supplementary Note 2**	Modelling of capacitance compensation in ACCEL.
**Supplementary Note 3**	Experimentally measured nonlinear response in ACCEL.
**Supplementary Note 4**	Experimental computing speed and energy efficiency of ACCEL with 1-layer digital computation.
**Supplementary Note 5**	Comparison between the comparator and ADC.
**Supplementary Note 6**	Weight-switching time of SRAM.
**Supplementary Note 7**	Design and simulation platform for EAC.
**Supplementary Note 8**	Calculations of the noise variance in ACCEL outputs.
**Supplementary Note 9**	Experimental computing speed and energy efficiency of ACCEL.
**Supplementary Table 1**	Benchmark of digital neural networks to compare with ACCEL.
**Supplementary Table 2**	Comparisons between the adopted comparator and the state-of-the-art 10-bit high-speed ADC fabricated with 180-nm CMOS process.
**Supplementary Table 3**	Experimentally measured latency for the output voltage of ACCEL to reach 20-dB signal-to-noise ratio (SNR) under different input light power.
**Supplementary Table 4**	Experimental computing performance of ACCEL in the all-analog mode.
**Supplementary Table 5**	Experimental computing performance of ACCEL connected with one digital layer.
**Supplementary Table 6**	Structures of digital neural networks with different layer numbers for comparison with ACCEL on 10-class MNIST classification.
**Supplementary Table 7**	Structures of digital neural networks with different layer numbers for comparisons with ACCEL for 3-class ImageNet classification.

Supplementary Note 1: The operation principle of ACCEL SRAM unit

The EAC weights of ACCEL are stored in the pixel-embedded SRAM macro shown in Extended Data Fig. 1a, and the detailed circuit structure of the SRAM unit is presented in Extended Data Fig. 1b. The SRAM write operation is performed by setting high write word-line (*WWL)* to turn on N-channel metal-oxide semiconductor (NMOS) transistor switches N_1 and N_2 in the SRAM unit, and setting the write bit-line *WBL* and *WBLB* voltage according to the data to be written. For *WBL*, the voltage should be set to supply voltage V_{DD} if the weight value to be written is +1, and set to ground voltage if the weight value to be written is -1. The voltage of the *WBLB* to be set is opposite to *WBL*.

The SRAM readout operation is performed in two phases. For example in Extended Data Fig. 1a, to read out w_0, the column-read switch S_{C0} is first turned on by setting signal *RCL[0]* high to connect the read bit-line *RBL[0]* to line D_{OUT}. Meanwhile, pre-charge switch S_{PRE} is turned on by setting signal *PRCH* low to charge the read bit-line *RBL[0]* to supply voltage V_{DD}. Pre-charge switch S_{PRE} is then turned off by setting signal *PRCH* high, and the read word-line signal *RWL[0]* is set high to turn on the readout NMOS transistor switch N_3 in the SRAM unit.

If $w_0 = +1$, the voltage of node *DB* (Extended Data Fig. 1b) in the SRAM unit would be ground voltage, and the NMOS transistor switch N_4 in the SRAM unit would be turned-off. In this case, there is no discharging path between read bit-line *RBL[0]* and the ground, so the voltage of D_{OUT}, which is connected to *RBL[0]*, stays at the supply voltage V_{DD}.

If $w_0 = -1$, the voltage of node *DB* in the SRAM unit would be supply voltage V_{DD}, and the NMOS transistor switch N_4 in the SRAM unit would be turned-on to discharge the read bit-line *RBL[0]*, as well as D_{OUT}, to the ground voltage. The voltage of D_{OUT} is buffered by two inverters to generate the final weight readout signals *Q* and *QB* to control the switches S2 and S3 in the pixel unit.

Supplementary Note 2: Modelling of capacitance compensation in ACCEL

EAC chip implements MAC operations directly utilizing the light-induced photocurrent, which accumulates on the two computing-line V_+/V_- to cause a voltage drop form a pre-charged voltage that indicates the MAC results. The capacitance load of V_+ and V_- line should be the same for the execution of standard MAC operation. However, the capacitance of the computing line V_+/V_- is dependent on the number of photodiodes connected to the line, which is determined by the number of positive/negative weights, and is also affected by the parasitic capacitance in the circuit. Therefore, we use one pair of positive/negative capacitance compensation module (P-CCM/N-CCM) demonstrated in Fig. 2f to tune the load capacitance of V_+ and V_- to the same value. The P-CCM/N-CCM contains 64 compensation photodiodes (PD_C) for capacitance tuning of V_+/V_-. The area of each of the PD_C is twice of the photodiode in the pixel, and the top of PD_C is covered with metal so that no light-induced photocurrent is generated. Hence, PD_C only serve as capacitor with capacitance value twice as the photodiode capacitance in the pixel. For the capacitance compensation, if V_+ has less load capacitance than V_-, certain amount of PD_C is connected V_+ to increase the load capacitance of V_+ to the same value of V_-, and vice versa. Since the difference of the number of pixel photodiodes connected to V_+ and V_- is an even number, the capacitance of PD_C is chosen as twice of the capacitance of the photodiode in the pixel. For the compensation of one group of MAC operations, a 6-bit register is employed to store the binary code which indicates the number of PD_C that should be connected to the computing line. As the weight parameters of each group of MAC operations are different, 16 group 6-bit registers are adopted to support up to 16 output for the EAC chip. For each

output, the 6-bit binary code of the corresponding output is selected with a 1 of 16 multiplexer (MUX), and decoded to thermometer-code to control 64 switches, each controlling the connection of one PD_C to the computing line.

Extended Data Fig. 1d illustrates the timing diagram of the capacitance compensation process, where the numbers of positive and negative weights are 490 and 534, respectively. The compensation-enable (*CE*) signal controls the connection between the computing lines V_+/V_- and the current source as shown in Fig. 2f. The process of the compensation is performed in multiple steps using a binary search strategy. Each step contains three operation phases: pre-charging, dis-charging, and comparing. First, the computing lines V_+ and V_- are pre-charged to supply voltage V_{DD} by enabling signal *RST* in the pixel. Signal *RST* is sequentially disabled and signal *CE* is set high to connect the current source to the computing lines. In this way, each of the V_+ line and V_- line is discharged by a current with the same value. After a pre-defined discharging time, the *CE* signal is set low and the voltage of V_+ and V_- are compared. In the case shown in Extended Data Fig. 1d, the voltage-drop on V_+ is greater than the voltage-drop on V_- in the first step, indicating load capacitance of V_+ is smaller than V_-. Therefore, extra amount of capacitance should be connected to V_+ to ensure the V_+ and V_- lines have the same value of load capacitance. A binary search algorithm is then adopted to determine the number of PD_C needed to be connected to the computing line V_+. According to the voltage comparison result, 32 PD_C are connected to V_+ at the end of the first step. In the second step, voltage drop on V_+ is smaller than voltage drop on V_-, indicating that the number of PD_C connected to V_+ should be reduced, and so the number is reduced to be 16. The above procedure is repeated until the voltage drop on V_+ and V_- line is the same at the end of the dis-charging operation. The capacitance compensation is performed during the system start-up, and once the capacitance compensation is finished, ACCEL is ready for computation.

Supplementary Note 3: Experimentally measured nonlinear response in ACCEL

We use the photoelectric nonlinearity between OAC and EAC as the nonlinearity in ACCEL. As the photodiode in ACCEL converts the complex optical field into electric current, the input amplitude of the optical field is nonlinear to the output current *I*. The optical field is composed of both the electric field whose amplitude is *E* and the magnetic field whose amplitude is *H*. The electric field and the magnetic field convert to each other constantly. We use the energy of the electric field to represent the energy of the optical field as common practice[1]. Then the relationship of photoelectric current *I* and the amplitude of the electric field of the light wave *E* can be described as[1,2]:

$$I = \frac{1}{2}\epsilon c\eta \frac{e}{h\nu} A E^2,$$

where ϵ is the dielectric constant; c is the speed of light; η is the responsivity of the photodiode depending on the characteristic of the photodiode; e is elementary charge; h is Planck's constant; ν is the frequency of light; A is the area of the photosensitive surface.

The photocurrent does not increase infinitely along with the increase of light power[3]. When the photocurrent of the photodiode saturates, the saturated photocurrent I_{sat}, input light power P_{sat} and the amplitude of electric field E_{sat} satisfy:

$$P_{sat} = \frac{I_{sat} h\nu}{\eta e}, E_{sat} = \sqrt{\frac{2 I_{sat} h\nu}{\epsilon c \eta e A}}.$$

In our demonstration, the response of the photodiode in ACCEL does not involve the saturate situation because ACCEL focuses on ultra-fast and low-exposure computation in vision tasks. For example, for incoherent situations, the power of sunlight can hardly reach the saturate power P_{sat}.

We measured the response to both coherent light at 532 nm (situations of Fig. 4-5) and incoherent white light (situations of Extended Data Fig. 4g-h and Supplementary Video 1) across a large intensity range to cover the exposure situations in the manuscript. White light can hardly reach extra high power in vision tasks in daily life, so it has a relatively small amplitude range (Extended Data Fig. 2a). For coherent light, we further enlarged the amplitude range with the laser (Changchun New Industries Optoelectronics Tech. Co., Ltd., MGL-III-532-300mW) to explore the response in a larger scope (Extended Data Fig. 2b). Both situations accord well with the theoretical quadric response and experimentally demonstrates the effective nonlinear response in ACCEL during all-analog computing.

Supplementary Note 4: Experimental computing speed and energy efficiency of ACCEL with 1-layer digital computation

The computing speed is calculated with the formula:

$$\text{Computing speed} = \frac{N_{op}}{t_{frame}} = \frac{N_o + N_e}{t_r + t_p + t_a + t_{AD} + t_{TPU}},$$

where N_{op} is the number of multiplication and adding operations ACCEL implements in one frame; t_{frame} is the time for ACCEL to process one frame; N_o is the number of operations implemented by OAC; N_e is the number of operations implemented by EAC; t_r is the reset time; t_p is the response time; t_a is the accumulating time; t_{AD} is the analog-to-digital data conversion time between ACCEL output and TPU input, and t_{TPU} is the time for the digital computing with TPU.

The number of operations implemented by ACCEL for one frame can be calculated as:

N_{op} (*3-class MNIST classification*) = $(2 \times 264^2 - 1) \times 1024 + (2 \times 1024 - 1) \times 16 + (2 \times 16 - 1) \times 3 = 1.43 \times 10^8$.

N_{op} (*10-class MNIST classification*) = $(2 \times 264^2 - 1) \times 1024 + (2 \times 1024 - 1) \times 16 + (2 \times 16 - 1) \times 10 = 1.43 \times 10^8$.

N_{op} (*time-lapse task*) = $(2 \times 448^2 - 1) \times 1024 + (2 \times 1024 - 1) \times 16 + (2 \times 48 - 1) \times 5/3 = 4.11 \times 10^8$.

The systemic energy efficiency is therefore calculated as:

$$\text{Energy efficiency}_{\text{sys}} = \frac{N_{op}}{E_{sys}} = \frac{N_o + N_e}{E_o + E_{cp} + E_{SRAM} + E_{control} + E_{AD} + E_{TPU}},$$

where E_{sys} is the systemic energy consumption of ACCEL; E_o is the laser energy; E_{cp} is the energy of the photocurrent to compute; E_{SRAM} is the energy of the SRAM to store, read and switch the weights; $E_{control}$ is the energy of control unit; E_{AD} is the energy consumption of the analog-to-digital data conversion, and E_{TPU} is the computing energy of the TPU.

We calculate the experimental performance of ACCEL for 3-class and 10-class image classification in Supplementary Table 5. The systemic computing speed of ACCEL for both 3-class and 10-class MNIST classification reaches 3.69×10^2 TOPS. The systemic energy efficiency of ACCEL for 3-class and 10-class image MNIST achieves 5.90×10^3 TOPS/W and 5.88×10^3 TOPS/W, respectively. The systemic computing speed of ACCEL for time-lapse tasks reaches 1.05×10^3 TOPS and the systemic energy efficiency achieves 4.22×10^3 TOPS/W.

Supplementary Note 5: Comparison between the comparator and ADC

ACCEL adopts the comparator shown in Extended Data Fig. 1e as an analog-to-digital interface instead of conventional ADC for time-lapse task. The comparator is composed of two back-to-back inverters that form a latch, and several switches for timing controlling. The timing diagram of the comparator is illustrated in Extended Data Fig. 1f. The comparator operates in three phases. First, the *RESET* signal is set high to clear the residual charges at sampling node S_+ and S_-. Then, the *RESET* signal is set low and the *SMP* signal is set high to sample the input voltages V_+ and V_- at the sampling node S_+ and S_- respectively. Finally, the *SMP* signal is set low and the *CMP_EN* signal is set high to compare the sampled voltages at S_+ and S_-. The voltage of the node with lower voltage is pulled down to ground voltage, and the voltage of the node with higher voltage is pulled up to supply voltage, which indicates the comparison result.

Since the comparator converts the input to a single-bit output, the conversion time and energy consumption of data conversion by comparator is much smaller than ADC, which samples the analog input signal and quantizes the input to multi-bit resolution digital data. For imaging application, the resolution of ADC is generally around 10-bit, and Supplementary Table 2 listed the performance of the comparator utilized in ACCEL and a state-of-the-art high-speed 10-bit ADC that is fabricated with 180 nm CMOS technology, the same as EAC. The conversion time and energy consumption of the comparator is respectively 3.81% and 1.21% of the ADC. Besides, ACCEL reduces the dimensionality of the input before the analog-to-digital interface from 224 × 224 (original image) to 16 (extracted analog features), so ACCEL reduces the latency and energy consumption of the analog-to-digital interface by 8.2×10^4 and 2.6×10^5 times.

Supplementary Note 6: Weight-switching time of SRAM

The weight-readout operation of SRAM is performed in two phases: pre-charge phase and readout phase (see Supplementary Note 1 for detailed principles of SRAM operation). To readout the data from SRAM, the control signal is first set low to reset the SRAM output node *Q* to 1, i.e., 1.8 V. Then the control signal is set high, and the on-chip controller generates the read word-line signal *RWL* to enable the readout process. If the data to be readout is 1, the output of SRAM stays at 1.8 V, and otherwise the output of SRAM is discharged to 0 V.

As the weight is binary, i.e., either -1 or 1 (corresponding to either V_- or V_+ lines in EAC), there are altogether four situations of the weight switching: from -1 to 1, from -1 to -1, from 1 to -1 and from 1 to 1. The experimentally measured weight switching time of the off-chip output signal is shown in Extended Data Fig. 7a-d. Since the process for weight changing by SRAM comprises two steps: 1) resetting the weight to 1; 2) changing to the new weight, the situation from 1 to 1 requires 0 ns. The measured weight signal indeed remained 1, i.e. the supply voltage 1.8 V (Extended Data Fig. 7a). Since SRAM output is a control signal that determines the switch in the pixel unit (Fig. 2h) to be turned on or off, the signal slightly below half supply voltage, i.e. 0.9 V, already means the switch is turned-off and does not necessarily have to be 0 V. Similarly, the signal slightly above half supply voltage, i.e. 0.9 V, already means the switch is turned-on, and does not necessarily have to be 1.8 V. Here we use 1.5 V and 0.3 V as the criteria instead of just 0.9 V to give an extra-secure upper-bound measurement. The off-chip output signal experimentally took 12.73 ns to switch from -1 to 1 (Extended Data Fig. 7b) and 13.54 ns to switch from 1 to -1 (Extended Data Fig. 7c). For the fourth situation: from -1 to -1, the internal signal changes from 0 V to 1.8 V as reset and then to 0 V. Because of the significant delay in the off-chip output signal, the output signal starts to drop

before it could actually reach 1.8 V (Extended Data Fig. 7d, the orange line). The measured latency for it to finish the -1 to 1 to -1 process is 12.97 ns. As this one is a complicated situation, we also labeled the latency for the signal to actually reach 0 V, which is 13.27 ns (Extended Data Fig. 7d, the green dashed vertical line).

As a result, we have measured all four situations of the weight switching by SRAM, and the time are 0 ns, 12.73 ns, 13.54 ns and 12.97 ns. Even the delayed off-chip output signal completes the weight switching within 14 ns, indicating the actual complete time inside the chip is within 14 ns. We also calculated the theoretical output signal with parameters provided by the foundry Semiconductor Manufacturing International Corporation (SMIC). The trends and calculation results correspond quite well with the measured results (Extended Data Fig. 7e-h) and also are all below 14 ns. We used 500 MHz as the clock frequency for ACCEL and assign 7 clock periods for reset time. Therefore, the reset operation of the computing-line and weight-switching of SRAM can be conducted simultaneously within the reset time of 14 ns.

Supplementary Note 7: Design and simulation platform for EAC

The design and pre-fabrication verification of the EAC chip is conducted with the Cadence design environment, via the 180 nm process design kit that includes the device model provided by Semiconductor Manufacturing International Corporation (SMIC), where the designed EAC chip is fabricated. We conduct post-simulation with the extracted post-end circuit netlist files from the chip layout to evaluate the speed performance of EAC. The required specification files for the extraction of post-end circuit netlist, including design rule checking (DRC), layout versus schematic (LVS) and parasitic electrical characteristic extraction (PEX), are also provided by SMIC.

Supplementary Note 8: Calculations of the noise variance in ACCEL outputs

Since the output voltage of ACCEL is inevitably affected by noise in the circuit, the voltage drop should be large enough to guarantee a detectable signal-to-noise ratio (SNR). The noise at the chip output mainly stems from $\sqrt{kT/C}$ noise, where k is the Boltzmann constant, T is thermodynamic temperature, and C is the load capacitance of the output signal line. The post-simulation with Cadence shows C is around 100 pF, indicating a noise level of 6.43 μV_{rms}. We select the time point where the voltage drop of EAC output voltage is 65 μV, thus providing a SNR of about 20 dB, to be the time point where the result is distinguishable enough, as presented in Fig. 6c.

Supplementary Note 9: Experimental computing speed and energy efficiency of ACCEL

The computing speed is calculated with the formula:

$$\text{Computing speed} = \frac{N_{op}}{t_{frame}} = \frac{N_o + N_e}{t_r + t_p + t_a},$$

where N_{op} is the number of multiplication and adding operations ACCEL implements in one frame; t_{frame} is the time for ACCEL to process one frame; N_o is the number of operations implemented by OAC; N_e is the number of operations implemented by EAC; t_r is the reset time; t_p is the response time and t_a is the accumulating time.

Although we used ACCEL with two-layer 400 × 400 OAC for 3-class ImageNet classification, the two OAC layers are two linearly connected matrix multiplication, without nonlinearity between. Therefore, we calculate the operation number in OAC as a matrix multiplication of a single 400 × 400 OAC layer as the minimum operation number. Then the minimum number of operations

implemented by ACCEL for one frame can be calculated as:

N_{op} (*3-class ImageNet classification*) = $(2 \times 400^2 - 1) \times 1024 + (2 \times 1024 - 1) \times 3 = 3.28 \times 10^8$.

N_{op} (*10-class MNIST classification*) = $(2 \times 264^2 - 1) \times 1024 + (2 \times 1024 - 1) \times 10 = 1.43 \times 10^8$

The systemic energy efficiency is therefore calculated as:

$$\text{Energy efficiency}_{\text{sys}} = \frac{N_{op}}{E_{sys}} = \frac{N_o + N_e}{E_o + E_{cp} + E_{SRAM} + E_{control}} ,$$

where E_{sys} is the systemic energy consumption of ACCEL; E_o is the laser energy; E_{cp} is the energy of the photocurrent to compute; E_{SRAM} is the energy of the SRAM to store, read and switch the weights and $E_{control}$ is the energy of control unit.

We calculate the experimental performance of ACCEL for 3-class and 10-class image classification in Supplementary Table 4. The systemic computing speed of ACCEL (10-class MNIST) reaches 5.95×10^2 TOPS and the systemic energy efficiency achieves 9.49×10^3 TOPS/W. The systemic computing speed of ACCEL (3-class ImageNet) reaches 4.55×10^3 TOPS and the systemic energy efficiency achieves 7.48×10^4 TOPS/W.

<table>
<tr><td colspan="6">Fig. 2b-c: Image reconstruction with OAC output (MNIST)</td></tr>
<tr><td colspan="3">Digital neural network</td><td colspan="3">ACCEL</td></tr>
<tr><td>Network structure</td><td>Neuron number in each layer</td><td>Nonlinearity</td><td>Network Structure</td><td>Neuron number in each layer</td><td>Nonlinearity</td></tr>
<tr><td>3-layer fully-connected digital NN</td><td>16 × 500, 500 × 500, 500 × 784</td><td>ReLU (between the first and second layer), tanh (between the second and third layer)</td><td colspan="3">Not applied.</td></tr>
<tr><td colspan="6">Fig. 2d: Classification with features compressed by OAC (MNIST)</td></tr>
<tr><td colspan="3">Digital neural network</td><td colspan="3">ACCEL</td></tr>
<tr><td>3-layer fully-connected digital NN</td><td>784 × 64, 64 × 64, 64 × 10</td><td>ReLU (between each two layers)</td><td colspan="3">Not applied.</td></tr>
<tr><td colspan="6">Fig. 3b: Image classification (MNIST)</td></tr>
<tr><td colspan="3">Digital neural network</td><td colspan="3">ACCEL</td></tr>
<tr><td rowspan="4">3-layer fully-connected digital NN</td><td rowspan="4">784 × 1024, 1024 × 16, 16 × 10</td><td rowspan="4">ReLU (between each two layers)</td><td>1-layer OAC</td><td>500 × 500</td><td>/</td></tr>
<tr><td>1-layer EAC</td><td>1024 × 10</td><td>/</td></tr>
<tr><td>1-layer OAC + 1-layer EAC</td><td>500 × 500, 1024 × 10</td><td>PD nonlinearity between OAC and EAC</td></tr>
<tr><td>1-layer OAC + 1-layer EAC + 1- layer digital NN</td><td>500 × 500, 1024 × 16, 16 × 10</td><td>PD nonlinearity (between OAC and EAC), ReLU (between EAC and digital NN)</td></tr>
<tr><td colspan="6">Fig. 3c: Image classification (Fashion-MNIST)</td></tr>
<tr><td colspan="3">Digital neural network</td><td colspan="3">ACCEL</td></tr>
<tr><td rowspan="2">3-layer fully-connected NN</td><td rowspan="2">784 × 1024, 1024 × 16, 16 × 10</td><td rowspan="2">ReLU (between each two layers)</td><td>1-layer OAC + 1-layer EAC</td><td>500 × 500, 1024 × 10</td><td>PD nonlinearity between OAC and EAC</td></tr>
<tr><td>1-layer OAC + 1-layer EAC + 1- layer digital NN</td><td>500 × 500, 1024 × 16, 16 × 10</td><td>PD nonlinearity (between OAC and EAC), ReLU (between EAC and digital NN)</td></tr>
<tr><td colspan="6">Fig. 3d: Image classification (3-class ImageNet)</td></tr>
</table>

Digital neural network			ACCEL		
3-layer fully-connected digital NN	65536 × 1024, 1024 × 16, 16 × 3	ReLU (between each two layers)	1-layer OAC + 1-layer EAC	400 × 400, 1024 × 3	PD nonlinearity between OAC and EAC
LeNet-5 (two convolutional layers with pooling, connected with three FC layers)	5 × 5 × 1 × 6 convolutional kernels, max-pooling, 5 × 5 × 6 × 16 convolutional kernels, max-pooling, 59536 × 120, 120 × 84, 84 × 3	ReLU (between each two layers)	6-layer OAC + 1-layer EAC	400 × 400, 400 × 400, 400 × 400, 400 × 400, 400 × 400, 400 × 400, 1024 × 3	PD nonlinearity between OAC and EAC
**Fig. 4f: Image classification (3-class ImageNet)**					
**Digital neural network**			**ACCEL**		
3-layer fully-connected digital NN	65536 × 1024, 1024 × 16, 16 × 3	ReLU (between each two layers)	1-layer OAC + 1-layer EAC	400 × 400, 1024 × 3	PD nonlinearity between OAC and EAC
LeNet-5 (two convolutional layers with pooling, connected with three FC layers)	5 × 5 × 1 × 6 convolutional kernels, max-pooling, 5 × 5 × 6 × 16 convolutional kernels, max-pooling, 59536 × 120, 120 × 84, 84 × 3	ReLU (between each two layers)	2-layer OAC + 1-layer EAC	400 × 400, 400 × 400, 1024 × 3	PD nonlinearity between OAC and EAC
**Fig. 5b: Video judgment**					
**Digital neural network**			**ACCEL**		
Not applied.			1-layer OAC + 1-layer EAC + 1-layer digital NN	448 × 448, 1024 × 16, 48 × 5	PD nonlinearity (between OAC and EAC), sign function (between EAC and digital NN)
**Fig. 6d-e: Image classification (3-class ImageNet)**					
**Digital neural network**			**ACCEL**		
Because it is too long for here, we listed it in Supplementary Table 7.			1-layer EAC	1024 × 3	/

			1-layer OAC + 1-layer EAC	400 × 400, 1024 × 3	PD nonlinearity between OAC and EAC
			2-layer OAC + 1-layer EAC	400 × 400, 400 × 400, 1024 × 3	PD nonlinearity between OAC and EAC
**Extended Data Fig. 5c: Image classification (MNIST) under low light**					
**Digital neural network**			**ACCEL**		
2-layer fully-connected digital NN	784 × 1024, 1024 × 10	ReLU (between each two layers)	1-layer OAC + 1-layer EAC	500 × 500, 1024 × 10	PD nonlinearity between OAC and EAC
**Extended Data Fig. 5d: Video judgment under low light**					
**Digital neural network**			**ACCEL**		
3-layer fully-connected digital NN	50176 × 1024, 1024 × 16, 48 × 5	ReLU (between each two layers)	1-layer OAC + 1-layer EAC + 1- layer digital NN	540 × 540, 1024 × 16, 48 × 5	PD nonlinearity (between OAC and EAC), sign function (between EAC and digital NN)
**Extended Data Fig. 9a: Image classification (10-class MNIST)**					
**Digital neural network**			**ACCEL**		
Because it is too long for here, we listed it in Supplementary Table 6.			1-layer EAC	1024 × 10	/
			1-layer OAC + 1-layer EAC	500 × 500, 1024 × 10	PD nonlinearity between OAC and EAC
			2-layer OAC + 1-layer EAC	500 × 500, 500 × 500, 1024 × 10	PD nonlinearity between OAC and EAC
			3-layer OAC + 1-layer EAC	500 × 500, 500 × 500, 500 × 500, 1024 × 10	PD nonlinearity between OAC and EAC
			4-layer OAC + 1-layer EAC	500 × 500, 500 × 500, 500 × 500, 500 × 500, 1024 × 10	PD nonlinearity between OAC and EAC
			5-layer OAC + 1-layer EAC	500 × 500, 500 × 500, 500 × 500, 500 × 500, 500 × 500, 1024 × 10	PD nonlinearity between OAC and EAC
			6-layer	500 × 500,	PD nonlinearity

	OAC + 1-layer EAC	500 × 500,<br>500 × 500,<br>500 × 500,<br>500 × 500,<br>500 × 500,<br>1024 × 10	between OAC and EAC
**Extended Data Fig. 9b: Image classification (3-class ImageNet)**			
**Digital neural network**	**ACCEL**		
Because it is too long for here, we listed it in Supplementary Table 7.	1-layer EAC	1024 × 3	/
	1-layer OAC + 1-layer EAC	400 × 400,<br>1024 × 3	PD nonlinearity between OAC and EAC
	2-layer OAC + 1-layer EAC	400 × 400,<br>400 × 400,<br>1024 × 3	PD nonlinearity between OAC and EAC
	3-layer OAC + 1-layer EAC	400 × 400,<br>400 × 400,<br>400 × 400,<br>1024 × 3	PD nonlinearity between OAC and EAC
	4-layer OAC + 1-layer EAC	400 × 400,<br>400 × 400,<br>400 × 400,<br>400 × 400,<br>1024 × 3	PD nonlinearity between OAC and EAC
	5-layer OAC + 1-layer EAC	400 × 400,<br>400 × 400,<br>400 × 400,<br>400 × 400,<br>400 × 400,<br>1024 × 3	PD nonlinearity between OAC and EAC
	6-layer OAC + 1-layer EAC	400 × 400,<br>400 × 400,<br>400 × 400,<br>400 × 400,<br>400 × 400,<br>400 × 400,<br>1024 × 3	PD nonlinearity between OAC and EAC

Supplementary Table 1 | Benchmark of digital neural networks to compare with ACCEL. PD: photodiode. OAC: optical analog computing. EAC: electronic analog computing. FC: fully-connected.

	Comparator	ADC (SOTA, for 10-bit)[5]
Time of per conversion	475.7 ps	12.5 ns
Energy of per conversion	393.5 fJ	32.63 pJ

Supplementary Table 2 | Comparisons between the adopted comparator and the state-of-the-art 10-bit high-speed ADC fabricated with 180-nm CMOS process. The data of the comparator is derived by circuit post-simulation with Cadence virtuoso tool according to model files provided by SMIC (Semiconductor Manufacturing International Corporation), where the EAC chip is fabricated with 180-nm standard CMOS process. The values of ADC are the latency and energy for 10-bit data. SOTA: state-of-the-art.

Input light power (μW)	Time for ACCEL output to reach 20-dB SNR (ns)
350	2.1
250	3.0
200	3.7
150	5.0
100	7.6
80	9.2
60	13.7
30	23.2
10	73.9

Supplementary Table 3 | Experimentally measured latency for the output voltage of ACCEL to reach 20-dB signal-to-noise ratio (SNR) under different input light power. The noise level of ACCEL output is 6.43 μV_{rms}, so ACCEL output has a SNR of 20 dB when the output voltage drops 65 μV. Since the photocurrent that causes the voltage drop is proportional to the input light power, the time it takes for ACCEL output voltage to drop by 65 μV is approximately inversely proportional to the input light power.

Symbol	Parameter	3-class ImageNet classification (ACCEL)	3-class ImageNet classification (EAC only)	10-class MNIST classification (ACCEL)
Neuron number to compute each frame	OAC	400 × 400	/	264 × 264
	EAC	1024 × 3	1024 × 3	1024 × 10
N_{op}	Operations per frame	3.28×10^8	6.14×10^3	1.43×10^8
t_r	Reset time	14 × 3 ns	14 × 3 ns	14 × 10 ns
$t_p + t_a$	Response time and accumulating time	10 × 3 ns	10 × 3 ns	10 × 10 ns
E_{cp}	Energy of the photocurrent to compute	0.01 nJ	0.01 nJ	0.04 nJ
E_o	Energy of the laser	3.40 nJ	3.40 nJ	11.77 nJ
E_{SRAM}	Energy of the SRAM to store	0.37 nJ	0.37 nJ	1.22 nJ
$E_{control}$	Energy of control unit	0.60 nJ	0.60 nJ	2.01 nJ
/	Systemic computing speed	4.55×10^3 TOPS	0.09 TOPS	5.95×10^2 TOPS
Energy efficiency_{sys}	Systemic energy efficiency	7.48×10^4 TOPS/W	1.40 TOPS/W	9.49×10^3 TOPS/W

Supplementary Table 4 | Experimental computing performance of ACCEL in the all-analog mode. The clock frequency in our ACCEL prototype is 500 MHz. The supply voltage of the computing module in EAC and SRAM is 1.8 V; the supply voltage of the control unit is 1.0 V; the measured average current of the computing module, control unit and SRAM are 89.15 µA, 8.38 mA and 2.83 mA respectively. Although we used ACCEL with two-layer 400 × 400 OAC for 3-class ImageNet classification, the two OAC layers are two linearly connected matrix multiplication, without nonlinearity between. Therefore, we calculate the operation number in OAC as a matrix multiplication of a single 400 × 400 OAC layer as the minimum operation number. We use the measured laser energy instead of the energy arriving at ACCEL as the energy of light.

Symbol	Parameter	3-class MNIST classification (ACCEL)	10-class MNIST classification (ACCEL)	Time-lapse task
Neuron number to compute each frame	First layer (OAC)	264 × 264	264 × 264	448 × 448
	Second layer (EAC)	1024 × 16	1024 × 16	1024 × 16
	Third layer (TPU)	16 × 3	16 × 10	48 × 5/3
N_{op}	Operations per frame	1.43×10^8	1.43×10^8	4.11×10^8
t_r	Reset time	14 × 16 ns	14 × 16 ns	14 × 16 ns
$t_p + t_a$	Response time and accumulating time	10 × 16 ns	10 × 16 ns	10 × 16 ns
t_{AD}	Analog-to-digital conversion time	0.20 × 16 ns*	0.20 × 16 ns*	0.48 × 16 ns†
t_{TPU}	Digital computing time	1.01 ps	3.37 ps	1.72 ps
E_{cp}	Energy of the photocurrent to compute	0.06 nJ	0.06 nJ	0.06 nJ
E_o	Energy of the laser	18.83 nJ	18.83 nJ	92.03 nJ
E_{SRAM}	Energy of the SRAM to store	1.96 nJ	1.96 nJ	1.96 nJ
$E_{control}$	Energy of control unit	3.22 nJ	3.22 nJ	3.22 nJ
E_{AD}	Energy of analog-to-digital conversion	92.80 pJ*	92.80 pJ*	6.30 pJ†
E_{TPU}	Energy of TPU	0.04 nJ	0.13 nJ	0.07 nJ
/	Systemic computing speed	3.69×10^2 TOPS	3.69×10^2 TOPS	1.05×10^3 TOPS
Energy efficiency$_{sys}$	Systemic energy efficiency	5.90×10^3 TOPS/W	5.88×10^3 TOPS/W	4.22×10^3 TOPS/W

Supplementary Table 5 | Experimental computing performance of ACCEL connected with one digital layer. *State-of-the-art high-speed 10-bit ADC[6]. †On-chip comparator in EAC chip. The clock frequency in our ACCEL prototype is 500 MHz. The supply voltage of the computing module in EAC and SRAM is 1.8 V; the supply voltage of the control unit is 1.0 V; the measured average current of the computing module, control unit and SRAM are 89.15 µA, 8.38 mA and 2.83 mA, respectively. We use the measured laser energy instead of the energy arriving at ACCEL as the energy of light.

MNIST (different layer numbers): Extended Data Fig. 9a						
**LeNet**	**Kernel size and neuron number in each layer (height × width × input channel × output channel)**					
**1st Conv**	5 × 5 × 1 × 10	5 × 5 × 1 × 10	5 × 5 × 1 × 10	5 × 5 × 1 × 10	5 × 5 × 1 × 10	5 × 5 × 1 × 10
**2nd Conv**	/	5 × 5 × 10 × 11	5 × 5 × 10 × 11	5 × 5 × 10 × 11	5 × 5 × 10 × 11	5 × 5 × 10 × 11
**3rd Conv**	/	/	5 × 5 × 11 × 12	5 × 5 × 11 × 12	5 × 5 × 11 × 12	5 × 5 × 11 × 12
**4th Conv**	/	/	/	5 × 5 × 12 × 15	5 × 5 × 12 × 15	5 × 5 × 12 × 15
**5th Conv**	/	/	/	/	5 × 5 × 15 × 18	5 × 5 × 15 × 18
**6th Conv**	/	/	/	/	/	5 × 5 × 18 × 21
**1st FC**	7290 × 120	7436 × 120	7500 × 120	8640 × 120	9522 × 120	10164 × 120
**2nd FC**	120 × 84	120 × 84	120 × 84	120 × 84	120 × 84	120 × 84
**3rd FC**	84 × 10	84 × 10	84 × 10	84 × 10	84 × 10	84 × 10
**Accuracy**	98.8%	99.1%	99.2%	99.3%	99.3%	99.3%
**Fully-connected**	**Neuron number in each layer (input neurons × output neurons)**					
**1st FC**	784 × 7500	784 × 7500	784 × 7500	784 × 7500	784 × 7500	784 × 7500
**2nd FC**	7500 × 10	7500 × 700	7500 × 1400	7500 × 1900	7500 × 2100	7500 × 2300
**3rd FC**	/	700 × 10	1400 × 700	1900 × 1400	2100 × 1900	2300 × 2100
**4th FC**	/	/	700 × 10	1400 × 700	1900 × 1400	2100 × 1900
**5th FC**	/	/	/	700 × 10	1400 × 700	1900 × 1400
**6th FC**	/	/	/	/	700 × 10	1400 × 700
**7th FC**	/	/	/	/	/	700 × 10
**Accuracy**	98.5%	98.6%	98.6%	98.6%	98.6%	98.6%

Supplementary Table 6 | Structures of digital neural networks with different layer numbers for comparison with ACCEL on 10-class MNIST classification. The size of the input image is 28 × 28. Each layer of the network is connected to a nonlinear ReLU layer, and the convolutional layer is connected to a pooling layer which has the pooling size of 2 and stride of 1 before ReLU. Each convolutional layer has padding of two pixels on each side. FC: fully-connected layer. Conv: convolutional layer.

3-class ImageNet (different layer numbers): Extended Data Fig. 9b, Fig. 6d-e						
**LeNet**	**Kernel size and neuron number in each layer (height × width × input channel × output channel)**					
**1st Conv**	5 × 5 × 1 × 2	5 × 5 × 1 × 3	5 × 5 × 1 × 3	5 × 5 × 1 × 3	5 × 5 × 1 × 3	5 × 5 × 1 × 3
**2nd Conv**	/	5 × 5 × 3 × 5	5 × 5 × 3 × 5	5 × 5 × 3 × 5	5 × 5 × 3 × 5	5 × 5 × 3 × 5
**3rd Conv**	/	/	5 × 5 × 5 × 10	5 × 5 × 5 × 10	5 × 5 × 5 × 10	5 × 5 × 5 × 10
**4th Conv**	/	/	/	5 × 5 × 10 × 16	5 × 5 × 10 × 16	5 × 5 × 10 × 15
**5th Conv**	/	/	/	/	5 × 5 × 16 × 20	5 × 5 × 15 × 18
**6th Conv**	/	/	/	/	/	5 × 5 × 18 × 22
**1st FC**	32768 × 120	20480 × 120	10240 × 120	15376 × 120	18000 × 120	18502 × 120
**2nd FC**	120 × 84	120 × 84	120 × 84	120 × 84	120 × 84	120 × 84
**3rd FC**	84 × 3	84 × 3	84 × 3	84 × 3	84 × 3	84 × 3
**Accuracy**	79.3%	84.0%	84.7%	87.3%	90.7%	90.7%
**Fully-connected**	**Neuron number in each layer (input neurons × output neurons)**					
**1st FC**	65536 × 90	65536 × 180	65536 × 270	65536 × 360	65536 × 450	65536 × 535
**2nd FC**	90 × 3	180 × 90	270 × 180	360 × 270	450 × 360	535 × 450
**3rd FC**	/	90 × 3	180 × 90	270 × 180	360 × 270	450 × 360
**4th FC**	/	/	90 × 3	180 × 90	270 × 180	360 × 270
**5th FC**	/	/	/	90 × 3	180 × 90	270 × 180
**6th FC**	/	/	/	/	90 × 3	180 × 90
**7th FC**	/	/	/	/	/	90 × 3
**Accuracy**	72.0%	76.7%	76.7%	78.0%	78.0%	78.0%

Supplementary Table 7 | Structures of digital neural networks with different layer numbers for comparisons with ACCEL for 3-class ImageNet classification. The size of the input image is 256 × 256. Each layer of the network is connected to a nonlinear ReLU layer, and the convolutional layer is connected to a pooling layer which has the pooling size of 2 and stride of 2 (1 when exceeding 3rd conv layers) before ReLU. Each convolutional layer has padding of two pixels on each side. FC: fully-connected layer. Conv: convolutional layer. NN: neural network.

References

1. Grant, I. S., & Phillips, W. R. *Electromagnetism*. (John Wiley & Sons, 2008)
2. Ohta, J. *Smart CMOS image sensors and applications*. (CRC Press, 2020).
3. Thorlabs Inc. Photodiode Saturation Limit and Noise Floor. https://www.thorlabs.com/images/TabImages/Photodetector_Lab.pdf (2015).
4. OMNIVISION. OMNIVISION Commercializes World's Smallest Pixel in New 200MP Image Sensor with Superior Low-light Performance for High-end Smartphones. https://www.ovt.com/press-releases/omnivision-commercializes-worlds-smallest-pixel-in-new-200mp-image-sensor-with-superior-low-light-performance-for-high-end-smartphones/ (2022).
5. Shen, Y., Zhu, Z., Liu, S. & Yang, Y. A Reconfigurable 10-to-12-b 80-to-20-MS/s bandwidth scalable SAR ADC. *IEEE Trans. Circuits Syst. I: Regular Papers* **65**, 51-60 (2018).
6. Guo, M., Mao, J., Sin, S. W., Wei, H. & Martins, R. P. A 29mW 5GS/s time-interleaved SAR ADC achieving 48.5dB SNDR with fully-digital timing-skew calibration based on digital-mixing. In *2019 Symposium on VLSI Circuits (VLSI)*, C76-C77 (IEEE, 2019)
